# Supplementary material for: Postcranial anatomy of Pissarrachampsa sera (Crocodyliformes, Baurusuchidae) from the Late Cretaceous of Brazil: insights on lifestyle and phylogenetic significance
Source: PeerJ. 2016 May 26;4:e2075. doi: 10.7717/peerj.2075 (PMC4888301; doi:10.7717/peerj.2075)
Supplement: Supplemental Information 1 [file peerj-04-2075-s001.docx]

Supplemental Information

**Postcranial anatomy of *Pissarrachampsa sera* (Crocodyliformes, Baurusuchidae) from the Late Cretaceous of Brazil: phylogenetic significance and comments on lifestyle**

Pedro L. Godoy^1*^, Mario Bronzati^2,3*^, Estevan Eltink^4^, Júlio C. de A. Marsola^4^, Giovanne M. Cidade^4^, Max C. Langer^4^, Felipe C. Montefeltro^5^

^1^ School of Geography, Earth and Environmental Sciences, University of Birmingham, Edgbaston, Birmingham, UK.

^2^Bayerische Staatssammlung für Paläontologie und Geologie, Munich, Germany.

^3^Ludwig-Maximilians Universität, Munich, Germany

^4^ Laboratório de Paleontologia de Ribeirão Preto, FFCLRP, Universidade de São Paulo, Ribeirão Preto, SP, Brazil.

^5^ Departamento de Biologia e Zootecnia, FEIS, Universidade Estadual Paulista, Ilha Solteira, Brazil.

* These authors contributed equally to this work.

**This file includes:**

1. Body size and mass estimations

2. Details of the phylogenetic analyses

2.1. Data Matrix

2.2. Character list

2.3. Results of the phylogenetic analysis

2.4. Exploratory analyses

3. Supplementary literature cited

**1 – Body size and mass estimations**

The total body lengths and masses were estimated using the regression equations published by Farlow *et al.* (2005).

**Table S1. List of selected femoral measurements for *Pissarrachampsa sera* (in millimeters)***.* **FL***,* femur length; **Fc**, femoral midshaft circumference; **Fdw**, femoral distal width; **Fdh**, femoral distal height; **Fpmx**, femoral maximum proximal diameter; **Fpmn**, femoral minimum proximal diameter.

| **FL** | **Fc** | **Fdw** | **Fdh** | **Fpmx** | **Fpmn** |
| --- | --- | --- | --- | --- | --- |
| 241 | 74 | 49 | 37 | 45 | 28 |

**Table S2. List of equations used for body mass and total length estimates (Farlow *et al.*, 2005). TL**, total length; **M**, body mass.

| **Total Length** | **Body Mass** |
| --- | --- |
| TL = 16.45 + (FL) 14.45 | log M = −5.72 + (log FL) 3.33 |
| log TL = 1.92 + (log Fc) 0.83 | log M = −3.25 (log Fc) 2.85 |
| log TL = 2.06 + (log Fdw) 0.82 | log M = −2.78 + (log Fdw) 2.80 |
| log TL = 2.24 + (log Fdh) 0.79 | log M = −2.23 + (log Fdh) 2.78 |
| log TL = 2.07+ (log Fpmx) 0.83 | log M = −2.72 + (log Fpmx) 2.80 |
| log TL = 2.33+ (log Fpmn) 0.79 | log M = −1.83 + (log Fpmm) 2.65 |

**Table S3. Body mass and total length estimates for *Pissarrachampsa sera* using Farlow *et al.* (2005) equations*.*** Total length in meters and body mass in kilograms.

| **Measurement** | **Total Length** | **Body Mass** |
| --- | --- | --- |
| **FL** | 3.4989 | 162.973 |
| **Fc** | 2.9612 | 119.484 |
| **Fdw** | 2.7923 | 89.650 |
| **Fdh** | 3.0122 | 134.772 |
| **Fpmx** | 2.7680 | 81.095 |
| **Fpmn** | 2.9734 | 101.151 |

**Table S4. Body mass and total length estimates for other baurusuchids using Farlow *et al.* (2005) equations*.*** Total length in meters and body mass in kilograms. **FL***,* femur length (in millimeters). Measurement data from Nascimento (2008) and Vasconcellos & Carvalho (2010)

| **Taxon** | **FL** | **Total Length** | **Body Mass** |
| --- | --- | --- | --- |
| *Baurusuchus albertoi* | 262 | 3.8024 | 215.250 |
| *Baurusuchus salgadoensis* | 265 | 3.8457 | 223.568 |

Additionally, we used the equations from Campione & Evans (2012) to estimate body mass in *Pissarrachampsa sera*. The femur-based equation is: logBM = 2.818*logCF - 0.417, where "BM" is body mass and "CF" is femoral circumference (74 millimeters for *Pissarrachampsa sera*). The body mass obtained for *Pissarrachampsa sera* was 70.875 kilograms.

**2 – Details of the phylogenetic analyses**

**2.1 – Data Matrix**

We scored 33 newly recognized postcranial features of *P. sera* in the original data matrix of Leardi et al. (2015a).

The character list is the same one of Leardi et al. (2015a). Following the original study, character 5 was excluded from the analysis and the following characters were set as additives: 1, 3, 6, 10, 23, 37, 43, 44, 45, 49, 65, 67, 69, 71, 73, 77, 79, 86, 90, 91, 96, 97, 105, 116, 126, 140, 142, 143, 149, 167, 182, 187, 193, 197, 226, 228, 279, 339, 356, 357, 364, 368, 401. The final data matrix is presented in nexus format (file available online: Phylogenetic matrix).

New characters scored for *P. sera* are: 85, 87, 93, 110, 113, 114, 116, 152, 153, 157, 323, 324, 331, 332, 333, 334, 335, 336, 337, 338, 339, 341, 342, 343, 344, 345, 419, 424, 425, 426, 427, 429, 439.

**2.2 – Character List**

Character 1 (modified from Clark, 1994: char. 1): + External surface of dorsal cranial bones: smooth (0), slightly grooved (1) and heavily ornamented with deep pits and grooves (2).

Character 2 (modified from Clark, 1994: char. 2): Skull expansion at orbits: gradual (0), or abrupt (1).

Character 3 (modified from Clark, 1994: char. 3): + Rostrum proportions: narrow oreinirostral (0), broad oreinirostral (1), nearly tubular (2), or platyrostral (3).

Character 4 (Clark, 1994: char. 4): Premaxilla participation in internarial bar: forming at least the ventral half (0), or with little participation (1).

Character 5 (Clark, 1994: char. 5): Premaxilla anterior to nares: narrow (0), or broad (1).

Character 6 (modified from Clark, 1994: char. 6): + External nares facing anterolaterally or anteriorly (0), dorsally not separated by premaxillary bar from anterior edge of rostrum (1), or dorsally separated by premaxillary bar (2).

Character 7 (Clark, 1994: char. 7): Palatal parts of premaxillae: do not meet posterior to incisive foramen (0), or meet posteriorly along contact with maxillae (1).

Character 8 (Clark, 1994: char. 8): Premaxilla-maxilla contact: premaxilla loosely overlies maxilla (i.e. posterodorsal process of the premaxilla overlaps anterodorsal surface of the maxilla) (0), or sutured together along a butt joint (1).

Character 9 (modified from Clark, 1994: char. 9): Ventrally opened notch on ventral edge of rostrum at premaxilla-maxilla contact: absent (0), present as a notch (1), or present as a large notch (2), or present as a notch that is closed ventrally (or largely constrained at its ventral edge) (3).

Character 10 (modified from Clark, 1994: char. 10): + Posterior palatal branches of maxillae anterior to palatines: do not meet (0), or meet extensively but posterior-most parts fail to meet (1), or meet entirely (2).

Character 11 (Clark, 1994: char. 11): Nasal contacts lacrimal (0), or does not contact (1).

Character 12 (Clark, 1994: char. 12): Lacrimal contacts nasal along medial edge only (0), or medial and anterior edges (1).

Character 13 (Clark, 1994: char. 13): Nasal contribution to narial border: yes (0), or no (1).

Character 14 (Clark, 1994: char. 14): Nasal-premaxilla contact: present (0), or absent (1).

Character 15 (modified from Clark, 1994: char. 15): Descending process of prefrontal: does not contact palate (0), or contacts palate (1).

Character 16 (Clark, 1994: char. 16): Postorbital-jugal contact: postorbital anterior to jugal (0), or postorbital medial to jugal (1), or postorbital lateral to jugal (2).

Character 17 (Clark, 1994: char. 17): Anterior part of the jugal with respect to posterior part: as broad (0), or twice as broad (1).

Character 18 (Clark, 1994: char. 18): Jugal bar beneath infratemporal fenestra: flattened (0), or rod-shaped (1).

Character 19 (Clark, 1994: char. 19): Quadratojugal dorsal process: narrow, contacting only a small part of postorbital (0), or broad, extensively contacting the postorbital (1).

Character 20 (Clark, 1994: char. 20): Frontal width between orbits: narrow, as broad as nasals (0), or broad, twice as broad as nasals (1).

Character 21 (Clark, 1994: char. 21): Frontals: paired (0), unpaired (1).

Character 22 (Clark, 1994: char. 22): Dorsal surface of frontal and parietal: flat (0), or with midline ridge (1).

Character 23 (modified from Clark, 1994: char. 23 by Buckley and Brochu, 1999: char. 81): + Parieto-postorbital suture: absent from dorsal surface of skull roof and supratemporal fossa (0), absent from dorsal surface of skull roof but broadly present within supratemporal fossa (1), or present within supratemporal fossa and on dorsal surface of skull roof (2).

Character 24 (Clark, 1994: char. 24): Supratemporal roof dorsal surface: complex (0), or dorsally flat “skull table” developed, with postorbital and squamosal with flat shelves extending laterally beyond quadrate contact (1).

Character 25 (modified from Clark, 1994: char. 25) Postorbital bar: sculpted (if skull sculpted) (0), or unsculpted (1).

Character 26 (modified from Clark, 1994: char. 26): Postorbital bar: transversely flattened (0), or cylindrical (1).

Character 27 (Clark, 1994: char. 27): Vascular opening in dorsal surface of postorbital bar: absent (0), or present (1).

Character 28 (modified from Clark, 1994: char. 28): Postorbital anterolateral process: absent or poorly developed (0), or well developed, long, and acute (1).

Character 29 (Clark, 1994: char. 29): Dorsal part of the postorbital: with anterior and lateral edges only (0), or with anterolaterally facing edge (1).

Character 30 (Clark, 1994: char. 30): Dorsal end of the postorbital bar broadens dorsally, continuous with dorsal part of postorbital (0), or dorsal part of the postorbital bar constricted, distinct from the dorsal part of the postorbital (1).

Character 31 (Clark, 1994: char. 31): Bar between orbit and supratemporal fossa broad and solid, with broadly sculpted dorsal surface if sculpture present (0), or bar narrow, sculpting restricted to anterior surface (1).

Character 32 (modified from Clark, 1994: char. 32): Parietal: with broad occipital portion (0), or without broad occipital portion (1).

Character 33 (Clark, 1994: char. 33) Parietal: with broad sculpted region separating fossae (0), or with sagittal crest between supratemporal fossae (1).

Character 34 (Clark, 1994: char. 34): Postparietal (dermosupraoccipital): a distinct element (0), or not distinct (fused with parietal?) (1).

Character 35 (Clark, 1994: char. 35): Posterodorsal corner of the squamosal: squared off, lacking extra “lobe” (0), or with unsculptured “lobe” (1).

Character 36 (modified from Clark, 1994: char. 36 and Riff, 2007: char. 36): Posterolateral process of squamosal: poorly developed and projected horizontally at the same level of the skull (0), elongated, thin, and posteriorly directed, not ventrally deflected (1), or elongated, posterolaterally directed, and ventrally deflected (2), or elongated and ventrally directed forming an angle of approximately 90 degrees with the skull roof (3), or posterodorsally deflected (4).

Character 37 (Clark, 1994: char. 37): + Palatines: do not meet on palate below the narial passage (0), form palatal shelves that do not meet (1), or meet ventrally to the narial passage, forming part of secondary palate (2).

Character 38 (Clark, 1994: char. 38): Pterygoid: restricted to palate and suspensorium, joints with quadrate and basisphenoid overlapping (0), or pterygoid extends dorsally to contact laterosphenoid and form ventrolateral edge of the trigeminal foramen, strongly sutured to quadrate and laterosphenoid (1).

Character 39 (modified from Clark, 1994: char. 39): Choanal opening: continuous with pterygoid ventral surface except for anterior and anterolateral borders (0), or opens into palate through a deep midline depression (choanal groove) (1).

Character 40 (Clark, 1994: char. 40): Palatal surface of pterygoids: smooth (0), or sculpted (1).

Character 41 (Clark, 1994: char. 41): Pterygoids posterior to choanae: separated (0), or fused (1).

Character 42 (modified from Clark, 1994: char. 42 and from Ortega et al., 2000: char. 139): Choanal opening size: moderately broad or narrow, equal or less than 30% the width between the lateral margins of the pterygoid flanges (0), or extremely broad approximately 50% the width between the lateral margins of the pterygoid flanges (1).

Character 43 (modified from Clark, 1994: char. 43): + Primary pterygoidean palate: forms posterior half of the choanal opening (0), or forms posterior, lateral and part of the anterior margin of the choana (1), or completely enclose choana (2).

Character 44 (modified from Pol and Norell, 2004 and Clark, 1994: char. 44): + Anterior edge of choanae situated between the suborbital fenestra (or anteriorly) (0), situated near the posterior edge of suborbital fenestra (1), or posterior to the suborbital fenestra (reaching in some cases the edge of pterygoid flange) (2).

Character 45 (Clark, 1994: char. 45): + Quadrate: without fenestrae (0), with single fenestrae (1), or with three or more fenestrae on dorsal and posteromedial surfaces (2).

Character 46 (Clark, 1994: char. 46): Posterior edge of quadrate: broad medial to tympanum, gently concave (0), or posterior edge narrow dorsal to otoccipital contact, strongly concave (1).

Character 47 (Clark, 1994: char. 47): Dorsal, primary head of quadrate articulates with: squamosal, otoccipital, and prootic (0), or with prootic and laterosphenoid (1).

Character 48 (Clark, 1994: char. 48): Ventrolateral contact of otoccipital with quadrate: very narrow (0), or broad (1).

Character 49 (Modified from Clark, 1994: char. 49): + Quadrate, squamosal, and otoccipital: do not meet to enclose cranioquadrate passage (0), enclose passage near lateral edge of skull (1), or meet lateral to the passage with otoccipital contacting the quadrate lateral to the posterior opening of the passage (2).

Character 50 (Clark, 1994: char. 50): Pterygoid ramus of quadrate: with flat ventral edge (0), or with deep groove along ventral edge (1).

Character 51 (Clark, 1994: char. 51): Ventromedial part of quadrate: does not contact otoccipital (0), or contacts otoccipital to enclose carotid artery and form passage for cranial nerves IX--XI (1).

Character 52 (Clark, 1994: char. 52): Eustachian tubes: not enclosed between basioccipital and basisphenoid (0), or entirely enclosed (1).

Character 53 (Clark, 1994: char. 53): Basisphenoid rostrum (cultriform process): slender (0), or dorsoventrally expanded (1).

Character 54 (Clark, 1994: char. 54): Basipterygoid process: prominent, forming movable joint with pterygoid (0), or basipterygoid process small or absent, with basisphenoid joint suturally closed (1).

Character 55 (modified from Clark, 1994: char. 55 by Ortega et al., 2000: char. 68): Basisphenoid ventral surface: shorter than the basioccipital (0), or wide and similar to, or longer in length than basioccipital (1).

Character 56 (modified from Clark, 1994: char. 56): Basisphenoid: exposed on ventral surface of braincase (0), or hidden from ventral surface by pterygoid flanges that extend posteriorly up to the level of the basioccipital-basisphenoid suture (1).

Character 57 (Clark, 1994: char. 57): Basioccipital: without well-developed bilateral tuberosities (0), or with large pendulous tubera (1).

Character 58 (Clark, 1994: char. 58): Otoccipital: without laterally concave descending flange ventral to subcapsular process (0), or with flange (1).

Character 59 (Clark, 1994: char. 59): Cranial nerves IX--XI: pass through common large foramen vagi in otoccipital (0), or cranial nerve IX passes medial to nerves X and XI in separate passage (1).

Character 60 (Clark, 1994: char. 60): Otoccipital: without large ventrolateral part ventral to paroccipital process (0), or with large ventrolateral part (1).

Character 61 (Clark, 1994: char. 61): Crista interfenestralis between fenestrae pseudorotunda and ovalis nearly vertical (0), or horizontal (1).

Character 62 (Clark, 1994: char. 62): Supraoccipital: forms dorsal edge of the foramen magnum (0), or otoccipitals broadly meet dorsal to the foramen magnum, separating supraoccipital from foramen (1).

Character 63 (Clark, 1994: char. 63): Mastoid antrum: does not extend into supraoccipital (0), or extends through transverse canal in supraoccipital to connect middle ear regions (1).

Character 64 (Clark, 1994: char. 64): Posterior surface of supraoccipital: nearly flat (0), or with bilateral posterior prominences (1).

Character 65 (modified from Clark, 1994: char. 65): + Palpebrals: absent (0), or one small palpebral present in orbit (1), or one large palpebral (2), or two large palpebrals (3).

Character 66 (Clark, 1994: char. 66): External nares: divided by a septum (0), or confluent (1).

Character 67 (Modified from Clark, 1994: char. 67): + Antorbital fenestra as large as orbit (0) or less than half the diameter of the orbit (1) or absent (2).

Character 68 (modified from Clark, 1994: char. 68 by Ortega et al., 2000: char. 41): Supratemporal fenestrae extension: relatively large, covering most of surface of skull roof (0), or relatively short, fenestrae surrounded by a flat and extended skull roof (1).

Character 69 (modified from Clark, 1994: char. 69): + Choanal groove: undivided (0), partially septated, with parts of the septum located dorsal to the lateral choanal margins (1), or completely septated, with septum leveled with the lateral margins of the choana along its entire length (2).

Character 70 (Clark, 1994: char. 70): Dentary: extends posteriorly beneath mandibular fenestra (0), or does not extend beneath fenestra (1).

Character 71 (modified from Clark, 1994: char. 71): + Lateral flange of retroarticular process: straight and directed ventrally forming an angle of approximately 90 degrees with the longitudinal axis of the mandibular ramus (0), directed posteroventrally or posteriorly, with posterior end slightly upturned (1), directed posteroventrally or posteriorly, with posterior end markedly recurved dorsally (2).

Character 72 (Clark, 1994: char. 72): Prearticular: present (0), or absent (1).

Character 73 (modified from Clark, 1994: char. 73): + Articular without medial process (0), with short process not contacting braincase (1), or with process articulating with otoccipital and basisphenoid (2).

Character 74 (Clark, 1994: char. 74): Dorsal edge of surangular: flat (0), or arched dorsally (1).

Character 75 (Clark, 1994: char. 75): Mandibular fenestra: present (0), or absent (1).

Character 76 (Clark, 1994: char. 76): Insertion area for M. pterygoideous posterior: does not extend onto lateral surface of angular (0), or extends onto lateral surface of angular (1).

Character 77 (modified from Clark, 1994: char. 77 and Brochu, 1999: char. 43): + Splenial involvement in symphysis in ventral view: not involved (0), involved slightly in symphysis forming up to 20% symphyseal length (1), or forming close to 30% of the symphyseal length (2), or extensively involved forming up to 50% of the symphyseal length and occupying more than the length of five alveoli (3).

Character 78 (Clark, 1994: char. 78): Posterior premaxillary teeth: similar in size to anterior teeth (0), or hypertrophied (1).

Character 79 (modified from Clark, 1994: char. 79): + Enlarged conical maxillary teeth: absent, no tooth size variation (0), one enlarged tooth (or enlarged wave of teeth) (1), or enlarged maxillary teeth curved in two waves (festooned) (2).

Character 80 (Clark, 1994: char. 80): Anterior dentary teeth opposite premaxilla-maxilla contact: no more than twice the length of other dentary teeth (0), or more than twice the length (1).

Character 81 (modified from Clark, 1994: char. 81): Dentary teeth posterior to tooth opposite premaxilla-maxilla contact: equal in size (0), or enlarged dentary teeth opposite to smaller teeth in maxillary toothrow (1).

Character 82 (modified from Clark, 1994: char. 82 by Ortega et al., 2000: char. 120): Anterior and posterior scapular edges: symmetrical in lateral view (0), anterior edge more strongly concave than posterior edge (1), or dorsally narrow with straight edges (2).

Character 83 (modified from Clark, 1994: char. 83 by Ortega et al., 2000: char. 121): Coracoid length: up to two-thirds of the scapular length (0), or subequal in length to scapula (1).

Character 84 (Clark, 1994: char. 84): Anterior process of ilium: similar in length to posterior process (0), or one-quarter or less of the length of the posterior process (1).

Character 85 (Clark, 1994: char. 85): Pubis: rodlike without expanded distal end (0), or with expanded distal end (1).

Character 86 (Clark, 1994: char. 86): + Pubis: forms anterior half of ventral edge of acetabulum (0), or pubis contacting the ilium but partially excluded from the acetabulum by the anterior process of the ischium (1), or pubis completely excluded from the acetabulum by the anterior process of the ischium (2).

Character 87 (Clark, 1994: char. 87): Distal end of femur: with large lateral facet for the fibula (0), or with very small facet (1).

Character 88 (Clark, 1994: char. 88): Fifth pedal digit: with phalanges (0), or without phalanges (1).

Character 89 (Clark, 1994: char. 89): Atlas intercentrum: broader than long (0), or as long as broad (1).

Character 90 (modified from Clark, 1994: char. 90): + Cervical neural spines: all anteroposteriorly large (0), only posterior ones rodlike (1), or all spines rodlike (2).

Character 91 (modified from Clark, 1994: char. 91; by Buscalioni and Sanz, 1988: char. 37; by Brochu, 1997: char. 7): + Hypapophyses in cervicodorsal vertebrae: absent (0); present only in cervical vertebrae (1), present in cervical and the first two dorsal vertebrae (2); present at least up to the third dorsal vertebra (3); or up to the fourth dorsal vertebrae; (4) or present beyond to the fourth dorsal vertebrae (5).

Character 92 (Clark, 1994: char. 92): Cervical vertebrae: amphicoelous or amphyplatian (0), or procoelous (1).

Character 93 (Clark, 1994: char. 93): Trunk vertebrae: amphicoelous or amphyplatian (0), or procoelous (1).

Character 94 (Modified from Clark, 1994: char. 94): First caudal vertebrae: amphicoelous or amphyplatian (0), biconvex (1), or opisthocoelous (2), or procoelous (3).

Character 95 (Clark, 1994: char. 95): Dorsal osteoderms: rounded or ovate (0), or rectangular, broader than long (1), or square (2), or rectangular, longer than broad (3).

Character 96 (modified from Clark, 1994: char. 96, and Brochu, 1997: char. 40): + Dorsal osteoderms: without articular anterior process (0), with a discrete convexity on anterior margin (1), or with a well-developed process located anterolaterally in dorsal parasagittal osteoderms (2).

Character 97 (modified from Clark, 1994: char. 97 by Ortega et al., 2000: chars. 107 and 108): + Rows of dorsal osteoderms: two parallel rows (0), more than two (1), or more than four with accessory ranges of osteoderms (sensu Frey, 1988) (2).

Character 98 (Modified from Clark, 1994: char. 98): Osteoderms: some or all imbricated (0), or sutured to one another (1), or not in contact (2).

Character 99 (Clark, 1994: char. 99): Tail osteoderms: dorsal only (0), or completely surrounded by osteoderms (1).

Character 100 (Clark, 1994: char. 100): Trunk osteoderms: absent from ventral part of the trunk (0), or present (1).

Character 101 (Clark, 1994: char. 101): Osteoderms: with longitudinal keels on dorsal surfaces (0), or without longitudinal keels (1).

Character 102 (Wu and Sues, 1996: char. 14): Jugal: participating in margin of antorbital fossa (0), or separated from it (1).

Character 103 (modified from Wu and Sues, 1996: char. 17): Mandibular symphysis in lateral view: shallow and tapering anteriorly (0), deep and tapering anteriorly (1), deep and anteriorly convex (2), or shallow and anteriorly convex (3).

Character 104 (modified from Wu and Sues, 1996: char. 23): Articular facet for quadrate condyle: wider than broad (0), or elongated, equal to or more than twice the length of the quadrate condyles (1).

Character 105 (modified from Wu and Sues, 1996: char. 24 and Wu et al., 1997: char. 124): + Jaw joint: placed at level with basioccipital condyle (0), below basioccipital condyle about above level of lower toothrow (1), or below level of toothrow (2).

Character 106 (modified from Wu and Sues, 1996: char. 27 and Ortega et al., 2000: char.133): Premaxillary teeth: five or more (0), four (1), three (2), or two (3).

Character 107 (modified from Wu and Sues, 1996: char. 29): Unsculptured region along alveolar margin on lateral surface of maxilla: absent (0), or present (1).

Character 108 (Wu and Sues, 1996: char. 30): Maxilla: with eight or more teeth (0), seven (1), six (2), five (3), or four teeth (4).

Character 109 (Wu and Sues, 1996: char. 33): Coracoid: without posteromedial or ventromedial process (0), with elongate posteromedial process (1), or distally expanded ventromedial process (2).

Character 110 (Wu and Sues, 1996: char. 40): Radiale and ulnare: short and massive (0), or elongate (1).

Character 111 (modified from Gomani, 1997: char. 4): Prefrontals anterior to orbits: elongated, oriented parallel to anteroposterior axis of the skull (0), or short and broad, oriented posteromedially-anterolaterally (1).

Character 112 (modified from Gomani, 1997: char. 32): Basioccipital and ventral part of otoccipital: facing posteriorly (0), or posteroventrally (1).

Character 113 (Buscalioni and Sanz, 1988: char. 35): Vertebral centra: cylindrical (0), or spool shaped (1).

Character 114 (modified from Buscalioni and Sanz, 1988: char. 39): Transverse process of posterior dorsal vertebrae dorsoventrally low and laminar (0), or dorsoventrally high (1).

Character 115 (Buscalioni and Sanz, 1988: char. 44): Number of sacral vertebrae: two (0), or more than two (1).

Character 116 (modified from Buscalioni and Sanz, 1988: char. 49): + Development and orientation of the rugose surface for the insertion of the M. iliotibialis that forms the supracetabular crest: lateromedially narrow and facing dorsally or slightly laterodorsally (0), lateromedially broad, forming a wide and markedly rugose attachment surface facing laterodorsally (1), or lateromedially broad and rugose that is highly deflected laterally forming a remarkably deep acetabulum (2).

Character 117 (Buscalioni and Sanz, 1988: char. 54): Proximal end of radiale expanded symmetrically, similarly to the distal end (0), or more expanded proximolaterally than proximomedially(1).

Character 118 (modified from Pol and Gasparini, 2009: char. 118): Lateral surface of the anterior region of surangular and posterior region of dentary: without a longitudinal depression (0), or with a deep, well-defined longitudinal groove (1).

Character 119 (modified from Ortega et al., 1996: char. 9): Ventral exposure of splenials along mandibular rami, posterior to the symphysis: absent (0), or present (1).

Character 120 (Modified from Ortega et al., 1996: char. 11, Ortega et al., 2000: char. 100, Andrade and Bertini 2008a: char. 132, and Turner and Sertich, 2010: char. 120): Tooth margins in posterior region of the toothrow: with denticulate carinae formed by homogeneous and symmetrical denticles with a sharp cutting edge (0), or without carinae or with smooth or crenulated carinae (1), or with tubercular, rounded denticles (anisomorph sensu Andrade and Bertini, 2008b) (2).

Character 121 (modified from Pol, 1999a: char. 133 and Ortega et al., 2000: char. 145): Lateral surface of anterior process of jugal: flat or convex (0), or bearing a longitudinal ridge or shelf running along its lateral surface and triangular depression underneath it (1).

Character 122 (Pol, 1999a: char. 134): Jugal: does not exceed the anterior margin of orbit (0), or exceeds margin (1).

Character 123 (Pol, 1999a: char. 135): Notch in premaxilla on lateral edge of external nares: absent (0), or present on the dorsal half of the external nares lateral margin (1).

Character 124 (Pol, 1999a: char. 136): Dorsal border of external nares: formed mostly by the nasals (0), or by both the nasals and premaxilla (1).

Character 125 (Pol, 1999a: char. 138): Posterodorsal process of premaxilla: absent (0), or present extending posteriorly wedging between maxilla and nasals (1).

Character 126 (Pol, 1999a: char. 139 and Ortega et al., 2000: char. 9): + premaxilla maxilla suture in palatal view, medial to alveolar region: anteromedially directed (0), sinusoidal, posteromedially directed on its lateral half and anteromedially directed along its medial region (1), or posteromedially directed (2).

Character 127 (modified from Pol, 1999a: char. 140): Nasal-premaxilla suture: laterally concave (0), or straight (1).

Character 128 (modified from Pol, 1999a: char. 141): Nasal lateral edges along the suture with the maxilla: nearly parallel (0), oblique to each other converging anteriorly (1), or oblique to each other diverging anteriorly (2).

Character 129 (Pol, 1999a: char. 143): Palatine anteromedial margin: exceeding the anterior margin of the palatal fenestrae extending anteriorly between the maxillae (0), or not exceeding the anterior margin of palatal fenestrae (1).

Character 130 (Pol, 1999a: char. 144): Dorsoventral height of jugal antorbital region respect to infraorbital region: equal or lower (0), or antorbital region more expanded than infraorbital region of jugal (1).

Character 131 (Pol, 1999a: char. 145): Maxilla-lacrimal contact: partially included in antorbital fossa (0), or completely included (1).

Character 132 (Pol, 1999a: char. 146): Lateral eustachian tube openings: located posteriorly to the medial opening (0), or aligned anteroposteriorly and dorsoventrally (1).

Character 133 (Pol, 1999a: char. 147): Anterior process of ectopterygoid: developed (0), or reduced-absent (1).

Character 134 (Pol, 1999a: char. 148): Posterior process of ectopterygoid: developed (0), or reduced-absent (1).

Character 135 (Pol, 1999a: char. 149 and Ortega et al., 2000: char. 13): Small neurovascular foramen located in the premaxillo-maxillary suture on the lateral surface of the rostrum (not for large mandibular teeth): absent (0), or present (1).

Character 136 (Modified from Pol, 1999a: char. 150): Jugal suture with quadratojugal directed: obliquely posteroventrally (0), or vertically as a blunt suture (1).

Character 137 (modified from Pol, 1999a: char. 151): Orientation of distal carina on upper posterior teeth and mesial carina on lower posterior teeth: oriented parallel to the longitudinal axis of skull (0), or obliquely oriented, at an angle of approximately 45 degrees with the longitudinal axis of the skull (1).

Character 138 (Pol, 1999a: char. 152): Large and aligned neurovascular foramina on lateral maxillary surface: absent (0), or present (1).

Character 139 (modified from Pol, 1999a: char. 153): External surface of maxilla: with a single plane facing laterally (0), or with ventral region facing laterally and dorsal region facing dorsolaterally (1).

Character 140 (Modified from Pol, 1999a: char. 154 and Ortega et al., 2000: char. 104): + Mid to posterior elements of the toothrows: crowns not compressed laterally, subcircular in cross section (0), or crowns slightly compressed laterally (1), or roots and crowns highly compressed laterally (2).

Character 141 (Pol, 1999a: char. 155): Posteroventral corner of quadratojugal: reaching the quadrate condyles (0), or not reaching the quadrate condyles (1).

Character 142 (modified from Pol, 1999a: char. 156): + Base of postorbital process of jugal: directed posterodorsally (0), or dorsally (1), or anterodorsally (2).

Character 143 (Pol, 1999a: char. 157): + Postorbital process of jugal: anteriorly placed (0), in the middle (1), or posteriorly positioned (2).

Character 144 (Pol, 1999a: char. 158 and Ortega et al., 2000: char. 36): Postorbitalectopterygoid contact: present (0), or absent (1).

Character 145 (Pol, 1999a: char. 161): Quadratojugal: not ornamented (0), or ornamented in the base (1).

Character 146 (Pol, 1999a: char. 162): Prefrontal-maxillary contact in the inner anteromedial region of orbit: absent (0), or present (1).

Character 147 (Pol, 1999a: char. 163): Basisphenoid: without lateral exposure (0), or with lateral exposure on the braincase (1).

Character 148 (modified from Pol, 1999a: char. 165): Quadrate process of pterygoids: well developed (0), or extremely short and poorly developed, failing to extend along the lateral margin of the basisphenoid and ending far away from the level of the lateral eustachian openings (1).

Character 149 (modified from Pol, 1999a: char. 166 and Ortega et al., 2000: char. 44): + Quadrate major axis directed: posteroventrally (0), ventrally (1), or anteroventrally (2).

Character 150 (Pol, 1999a: char. 167): Quadrate distal end: with only one plane facing posteriorly (0), or with two distinct faces in posterior view divided by a ridge, a posterior one and a medial one bearing the foramen aerum (1).

Character 151 (Pol, 1999a: char. 168): Anteroposterior development of neural spine in axis: well developed covering all the neural arch length (0), or poorly developed, located over the posterior half of the neural arch (1).

Character 152 (Pol, 1999a: char. 169): Prezygapophyses of axis: not exceeding anterior edge of neural arch (0), or exceeding the anterior margin of neural arch (1).

Character 153 (Pol, 1999a: char. 170): Postzygapophyses of axis: well developed, curved laterally (0), or poorly developed (1).

Character 154 (modified from Pol, 1999b: char. 212): Shape of dentary symphysis in ventral view: tapering anteriorly forming an angle (0), U-shaped, smoothly curving anteriorly (1), or lateral edges longitudinally oriented, convex anterolateral corner, and extensive transversely oriented anterior edge (2).

Character 155 (Pol, 1999b: char. 213): Unsculpted region in the dentary below the tooth row: absent (0), or present (1).

Character 156 (Buckley and Brochu, 1999: char. 102): Surangular forms only the lateral wall of glenoid fossa and quadratojugal lacks an articular condyle (0) or surangular forms approximately one-third of the glenoid fossa and quadratojugal bears an articular condyle (1).

Character 157 (modified from Buckley and Brochu, 1999: char. 102): Anterior margin of femur at the area of insertion of M. puboischiofemoralis internus 1 (PIFI1) and M. caudofemoralis longus (CFL): anterior margin of femur linear (0), or bearing a distinct flange (that projects anteriorly the insertion areas for these muscles) and a marked concavity above this region (1).

Character 158 (modified from Buckley and Brochu, 1999: char. 105): Dentary smooth lateral to seventh alveolus (0), or with lateral concavity for the reception of the enlarged maxillary tooth (1).

Character 159 (modified from Ortega et al., 1995: char. 1 and Buckley and Brochu, 1999: char. 107): Dorsal edge of dentary slightly concave or straight and subparallel to the longitudinal axis of skull (0), straight with an abrupt dorsal expansion, being straight posteriorly (1), with a single dorsal expansion and concave posterior to this (2), or sinusoidal, with two concave waves (3).

Character 160 (modified from Ortega et al., 1995: char. 2 and Buckley and Brochu, 1999: char. 108): Dentary compression and lateroventral surface anterior to mandibular fenestra: compressed and vertical (0), or not compressed and convex (1).

Character 161 (modified from Ortega et al., 1995: char. 7 and Buckley and Brochu, 1999: char. 110): Splenial: thin posterior to symphysis (0), or splenial robust dorsally posterior to symphysis, being much broader than the lateral alveolar margin of the dentary at the same region (1).

Character 162 (Ortega et al., 1996: char. 13 and Buckley et al., 2000: char. 117): Cheek teeth: not constricted at base of crown (0), or constricted (1).

Character 163 (Ortega et al., 2000: char. 10): Ventral edge of premaxilla located: at the same height that ventral edge of maxilla (0), or located deeper, with the dorsal contour of anterior part of dentary strongly concave (1).

Character 164 (modified from Ortega et al., 2000: char. 19): Maxillary dental implantation: teeth in isolated alveoli (0), or located on a dental groove (1).

Character 165 (Ortega et al., 2000: char. 24): Caudal tip of nasals: converge at sagittal plane forming a transversely straight or a shallow posteriorly concave arch along their posterior margins (0), or caudally separated by an anterior acute sagittal projection of frontals (1).

Character 166 (Ortega et al., 2000: char. 33): Relative length between squamosal and postorbital: squamosal is longer (0), or postorbital is longer (1).

Character 167 (modified from Ortega et al., 2000: character 34): + Jugal portion of postorbital bar: flushes with lateral surface of jugal (0), anteriorly continuous but posteriorly inset (1), or medially displaced and a ridge separates postorbital bar from lateral surface of jugal (2).

Character 168 (modified from Ortega et al., 2000: char. 42): Outer surface of squamosal along the site of attachment of ear valve groove: laterodorsally oriented and extensive (0), or reduced and vertically oriented (1).

Character 169 (Ortega et al., 2000: char. 47): Quadratojugal spine at caudal margin of infratemporal fenestra: absent (0), or present (1).

Character 170 (modified from Ortega et al., 2000: char. 53): Quadrate condyles with poorly developed intercondylar groove (0), or medial condyle expands ventrally, being separated from the lateral condyle by a deep intercondylar groove (1).

Character 171 (Ortega et al., 2000: char. 62): Exposure of supraoccipital in skull roof: absent (0), or present (1).

Character 172 (Ortega et al., 2000: char. 70): Nasal participation in antorbital fenestra: yes (0), or no (1).

Character 173 (Ortega et al., 2000: char. 75): Anterior opening of temporo-orbital in dorsal view exposed (0), or hidden in dorsal view and overlapped by squamosal rim of supratemporal fossa (1).

Character 174 (modified from Ortega et al., 2000: char. 90): Foramen intermandibularis oralis: small or absent (0), or big and slot like, with their anteroposterior length being approximately or more than 50% of the depth of the splenial (1).

Character 175 (modified from Ortega et al. 2000: char 98): Coronoid size: short and located below the dorsal edge of the mandibular ramus (0), or anteriorly extended with posterior region elevated at the dorsal margin of the mandibular ramus (1).

Character 176 (Ortega et al., 2000: char. 101): Width of root of teeth respect to crown: much narrower (0), or subequal or wider (1).

Character 177 (Ortega et al., 2000: char. 109): Gap in cervico-thoracic dorsal armor: absent (0) or present (1).

Character 178 (Ortega et al., 2000: char. 130): Lateral contour of snout in dorsal view: straight (0) or sinusoidal (1).

Character 179 (Modified from Ortega et al., 2000: char. 138): Pterygoid flanges: laminar and with anteroposteriorly broad lateral end (0) or lateromedially elongated with anteroposteriorly short lateral end (1), or lateromedially short and with narrow lateral end (2).

Character 180 (modified from Ortega et al., 2000: char. 146): Ectopterygoid medial process: single, projected posteriorly on the ventral or lateral surface of the pterygoid flanges (0) or forked, with an accessory anteromedial branch reaching the palatine and forming part of the lateral margin of the choanal opening (1).

Character 181 (modified from Ortega et al., 2000: char. 157): Skull roof: rectangular shaped in dorsal view (0), or trapezoidal shape (1).

Character 182 (Ortega et al., 2000: char. 30): + Prefrontal pillars when integrated in palate: pillars transversely expanded (0), transversely expanded in their dorsal part and columnar (or slightly anteroposteriorly elongated) in the ventral end (1), or longitudinally expanded in their dorsal part and columnar ventrally (2).

Character 183 (Ortega et al., 2000: char. 21): Ventral edge of maxilla in lateral view: straight or convex (0), or sinusoidal (1).

Character 184 (modified from Ortega et al., 2000: char. 156): Position of first enlarged maxillary teeth: second or third alveoli (0), or fourth or fifth (1).

Character 185 (Pol and Apesteguia, 2005: char. 180): Splenial-dentary suture at symphysis on ventral surface: v-shaped (0), or transversal (1).

Character 186 (Pol and Apesteguia, 2005: char. 181): Posterior peg at the posterior edge of the mandibular symphysis: absent (0), or present (1).

Character 187 (Pol and Apesteguia, 2005: char. 182): Posterior ridge on glenoid fossa of articular: present (0), or absent (1).

Character 188 (modified from Gomani, 1997: char. 46 and Buckley et al., 2000: char. 113): Cusps of posterior teeth: unique apical cusp (0), at least three cusps, a major central cusp with smaller cusps arranged along the mesial and distal margins of the crown (1).

Character 189 (Pol and Apesteguia, 2005: char. 184): Dorsal surface of mandibular symphysis: flat or slightly concave (0), or strongly concave and narrow, trough shaped (1).

Character 190 (Pol and Apesteguia, 2005: char. 185): Medial surface of splenials posterior to symphysis: flat or slightly convex (0), or markedly concave (1).

Character 191 (modified from Pol and Apesteguia, 2005: char. 186): Choanal septum shape: narrow vertical bony sheet (0), or T-shaped bar expanded ventrally (1).

Character 192 (Pol and Norell, 2004a: char. 164): Cross section of distal end of quadrate: mediolaterally wide and anteroposteriorly thin, being approximately three times as wide as long (0), or subquadrangular or up to twice as broad as anteroposteriorly long (1).

Character 193 (modified from Pol and Apesteguia, 2005: char. 188): + Lateral surface of dentaries below alveolar margin, at mid to posterior region of tooth row: vertically oriented, continuous with rest of lateral surface of the dentaries (0), or flat surface facing laterally or laterodorsally but divided by a ridge from rest of the lateral surface of the dentaries (1), or posterior region of alveolar facing dorsally, forming a broad alveolar shelf that is strongly inset medially from the lateral surface of the dentaries (2).

Character 194 (Pol and Norell, 2004a: char. 165): Palatine-pterygoid contact on anterior region of palate: palatines overlie pterygoids (0), or palatines firmly sutured to pterygoids (1).

Character 195 (Pol et al., 2004: char. 164): Ectopterygoid main axis oriented: laterally or slightly anterolaterally (0), or anteriorly, subparallel to the skull longitudinal axis (1).

Character 196 (Wu et al., 1997: char. 103): Squamosal descending process: absent (0), or present (1).

Character 197 (modified from Wu et al., 1997: char. 105): + Development of distal quadrate body ventral to otoccipital-quadrate contact: distinct (0), incipiently distinct (1), or indistinct (2).

Character 198 (Modified from Wu et al., 1997: char. 106): Posterior margin of pterygoid flanges: thin and laminar (0), or dorsoventrally thick, with pneumatic spaces (1).

Character 199 (Wu et al., 1997: char. 108): Postorbital participation in infratemporal fenestra: almost or entirely excluded (0), or bordering infratemporal fenestra (1).

Character 200 (Wu et al., 1997: char. 109): Palatines: form margin of suborbital fenestra (0), or excluded from margin of suborbital fenestra (1).

Character 201 (Wu et al., 1997: char. 110): Angular posterior to mandibular fenestra: widely exposed on lateral surface of mandible (0), or shifted to the ventral surface of mandible (1).

Character 202 (Wu et al., 1997: char. 112): Posteroventral edge of mandibular ramus: straight or convex (0), or markedly deflected (1).

Character 203 (modified from Wu et al., 1997: char. 119): Quadrate process of pterygoid in ventral view: narrow (0), or broad (1).

Character 204 (Wu et al., 1997: char. 121): Pterygoids: not in contact anterior to basisphenoid on palate (0), or pterygoids in contact (1).

Character 205 (modified from Wu et al., 1997: char. 122): Olecranon: well developed (0), or reduced or absent (1).

Character 206 (Wu et al., 1997: char. 123): Cranial table width respect to ventral portion of skull: as wide as ventral portion (0), or narrower than ventral portion of skull (1).

Character 207 (Wu et al., 1997: char. 127): Depression on posterolateral surface of maxilla: absent (0), or present (1).

Character 208 (modified from Wu et al., 1997: char. 128): Anterior palatal fenestra: absent (0), or present (1).

Character 209 (Pol and Norell, 2004a: char. 179): Paired ridges located medially on ventral surface of basisphenoid: absent (0), or present (1).

Character 210 (Pol et al., 2004a: char. 179): Ventral margin of infratemporal bar of jugal: straight (0), or dorsally arched (1).

Character 211 (Pol and Norell, 2004a: char. 180): Posterolateral end of quadratojugal: acute or rounded, tightly overlapping the quadrate (0), or with sinusoidal ventral edge and wide and rounded posterior edge slightly overhanging the lateral surface of the quadrate (1).

Character 212 (Pol and Norell, 2004a : char. 181): Orientation of quadrate body distal to otoccipital-quadrate contact in posterior view: ventrally (0), or ventrolaterally (1).

Character 213 (Gasparini et al., 1993: char. 3): Wedge-like process of the maxilla in lateral surface of premaxilla-maxilla suture: absent (0), or present (1).

Character 214 (Pol and Norell, 2004b: char. 181): Palpebrals: separated from the lateral edge of the frontals (0), or extensively sutured to each other and to the lateral margin of the frontals (1).

Character 215 (Pol and Norell, 2004b: char. 182): External surface of ascending process of jugal: exposed laterally (0), or exposed posterolaterally (1).

Character 216 (Pol and Norell, 2004b: char. 183): Longitudinal ridge on lateral surface of jugal below infratemporal fenestra: absent (0), or present (1).

Character 217 (Pol and Norell, 2004b: char. 184): Oblique ridges on the dorsal surface of posterolateral region of squamosal: without ridges (0), or with three curved ridges oriented longitudinally (1).

Character 218 (Pol and Norell, 2004b: char. 185): Ridge along dorsal section of quadratequadratojugal contact: absent (0), or present (1).

Character 219 (modified from Pol and Norell, 2004b: char. 186): Sharp ridge on the surface of the angular: absent (0), or present on the ventral-most margin (1), or present along the lateral surface (2).

Character 220 (Pol and Norell, 2004b: char. 187): Longitudinal ridge along the dorsolateral surface of surangular: absent (0), or present (1).

Character 221 (Pol and Norell, 2004b: char. 188): Dorsal surface of osteoderms ornamented with anterolaterally and anteromedially directed ridges (fleur de lys pattern of Osmolska et al., 1997): absent (0), or present (1).

Character 222 (Pol and Norell, 2004b: char. 189): Cervical region surrounded by lateral and ventral osteoderms sutured to the dorsal elements: absent (0), or present (1).

Character 223 (Pol and Norell, 2004b: char. 190): Appendicular osteoderms: absent (0), or present (1).

Character 224 (Ortega et al., 2000: character 72): Supratemporal fenestra: present (0), or absent (1).

Character 225 (modified from Pol and Apesteguia, 2005: char. 220): Flat ventral surface of internal nares septum: parallel sided (0), or tapering anteriorly (1), or tapering posteriorly (2).

Character 226 (Pol and Apesteguia, 2005: char. 221): + Perinarial fossa: restricted extension (0), extensive, with a distinctly concave surface facing anteriorly (1), or large concave surface facing anteriorly, projecting anteroventrally from the external nares opening toward the alveolar margin (2).

Character 227 (Sereno et al., 2001: char. 67): Premaxillary palate circular paramedian depressions: absent (0), or present located anteriorly on the premaxilla (1).

Character 228 (modified from Pol and Apesteguia, 2005: char. 223): + Posterolateral region of nasals: flat surface facing dorsally and well separated from the anterodorsal corner of the orbit (0), or expanded posterolaterally reaching the anterior tip of the palpebral facet but limited to the dorsal surface of the skull (1), or well developed posterolateral process that deflects ventrally, forming part of the lateral surface of the snout (2).

Character 229 (Zaher et al., 2006: char. 193): Ventral half of the lacrimal: extending ventroposteriorly widely contacting the jugal (0), or tapering ventroposteriorly, does not contact or contacts the jugal only slightly (1).

Character 230 (Zaher et al., 2006: char. 194): Large foramen on the lateral surface of jugal, near its anterior margin: absent (0), or present (1).

Character 231 (modified from Zaher et al., 2006: char. 195): Procumbent premaxillary alveoli absent (0) or present (1).

Character 232 (modified from Martinelli, 2003: char. 36, Zaher et al., 2006: char. 196, and Turner, 2004: char. 119): Posterolateral end of palatines, completely sutured to the pterygoids (0) or project posterolaterally as rodlike palatine bars (1).

Character 233 (Modified from Zaher et al., 2006: char. 197): Participation of ectopterygoid in the lateral margin of the choanal opening: absent or reduced, less than one third of this margin (0), or extensive forming half or more of this margin (1).

Character 234 (Pol and Norell, 2004a: char. 183): Choanal opening: opened posteriorly and continuous with pterygoid surface (0), or closed posteriorly by an elevated wall formed by the pterygoids (1).

Character 235 (Modified from Zaher et al., 2006: char. 198): Ectopterygoid width at its contact with the ventral surface of pterygoid flanges: lateromedially thin process (0), or lateromedially expanded with respect to the shaft of the ectopterygoid, covering approximately the lateral half of the ventral surface of the pterygoid flanges (1).

Character 236 (Pol and Gasparini, 2009: char. 236): Evaginated maxillary alveolar edges: absent (0), or present as a continuous sheet (1), or present as discrete evaginations at each alveoli (2).

Character 237 (Pol and Gasparini, 2009: char. 237): Foramen in perinarial depression of premaxilla: absent (0), or present (1).

Character 238 (Sereno et al., 2001: char. 27): Frontal anterior ramus with respect to tip of prefrontal: ending posteriorly (0), or ending anteriorly (1).

Character 239 (modified from Sereno et al., 2001: char. 68): Premaxillary anterior alveolar margin orientation: vertical (0), or inturned (1).

Character 240 (Sereno et al., 2001: char. 69): Premaxillary tooth row orientation: arched posteriorly from midline (0), or angled posterolaterally, at 120 degree angle (1).

Character 241(Sereno et al., 2001: char. 70): Last premaxillary tooth position relative to tooth row: anterior (0), or anterolateral (1).

Character 242: Sutural contact between premaxilla and maxilla on dorsal surface of rostrum posterior to external nares: Premaxillae posterior tip V-shaped, wedging between maxillae (0), or posterior end of premaxillae W-shaped with the anterior tip of maxillae wedging between premaxillae (1).

Character 243 (modified from Brochu, 1999: char. 108 and from Pol and Gasparini, 2009: char. 243): Maxilla-palatine suture: palatine anteriorly rounded (0), or palatine anteriorly pointed (1), or palatine anterior end slightly invaginated (2), or palatine anterior end divided by a narrow and pointed process of the palatal branches of maxilla (3).

Character 244 (Pol and Gasparini, 2009: char. 244): Lateral surface of postorbital bar: formed by postorbital and jugal (0), or only by postorbital (1).

Character 245 (Pol and Gasparini, 2009: char. 245): Enlarged foramen at anterior end of surangular groove: absent (0), or present (1).

Character 246 (Pol and Gasparini, 2009: char. 246): Shape of antorbital fossa: subcircular or subtriangular (0), or elongated, low, and oriented obliquely (1).

Character 247 (Pol and Gasparini, 2009: char. 247): Prefrontal lateral development: reduced (0), or enlarged, extending laterally over the orbit (1).

Character 248 (Pol and Gasparini, 2009: char. 248): Foramen for the internal carotid artery: reduced, similar in size to the openings for cranial nerves IX-XI (0), or extremely enlarged (1).

Character 249 (Pol and Gasparini, 2009: char. 249): Squamosal posterolateral region, lateral to paroccipital process: narrow (0), or bearing a subcircular flat surface (1).

Character 250 (Pol and Gasparini, 2009: char. 250): Posteromedial branch of squamosal oriented: transversely (0), or posterolaterally (1).

Character 251 (Pol and Gasparini, 2009: char. 251): Dorsal margin of squamosal occipital flange: straight (0), or dorsally concave (1).

Character 252 (Pol and Gasparini, 2009: char. 252): Sculpture in external surface of rostrum: absent (0), or present (1).

Character 253 (Pol and Gasparini, 2009: char. 253): Longitudinal depressions on palatal surface of maxillae: absent (0), or present (1).

Character 254 (Pol and Gasparini, 2009: char. 254): Angle between medial and anterior margins of supratemporal fossa: approximately 90 degrees (0), or approximately 45 degrees (1).

Character 255 (Pol and Gasparini, 2009: char. 255): Transverse process of sacral vertebrae directed: laterally (0), or markedly deflected ventrally (1).

Character 256 (Pol and Gasparini, 2009: char. 256): Prefrontal and lacrimal around orbits: forming flat rims (0), or evaginated, forming elevated rims (1).

Character 257 (Pol and Gasparini, 2009: char. 257): Nasal bones: paired (0), or partially or completely fused (1).

Character 258 (Brochu, 1997: char. 3): Posterior half of axis neural spine wide (0) or narrow (1).

Character 259 (Brochu, 1997: char. 19): Axial hypapophysis without (0) or with (1) deep fork.

Character 260 (Brochu, 1997: char. 27): Olecranon process of ulna narrow and subangular (0) or wide and rounded (1).

Character 261 (Brochu, 1997: char. 29): M. teres major and M. dorsalis scapulae insert separately on humerus; scars can be distinguished dorsal to deltopectoral crest (0) or insert with common tendon; single insertion scar (1).

Character 262 (modified from Brochu, 1997: char. 53): Anterior dentary alveoli project anterodorsally or weakly procumbent (0) or strongly procumbent (1).

Character 263 (Brochu, 1997: char. 84): Dorsal and ventral rims of squamosal groove for external ear valve musculature parallel (0) or squamosal groove flares anteriorly (1).

Character 264 (Brochu, 1997: char. 91): Ectopterygoid abuts maxillary toothrow (0) or maxilla broadly separates ectopterygoid from maxillary toothrow (1).

Character 265 (Brochu, 1997: char. 92): Shallow fossa at anteromedial corner of supratemporal fenestra (0) or no such fossa; anteromedial corner of supratemporal fenestra smooth (1).

Character 266 (modified from Brochu, 1997: char. 103): Lateral margins of frontal: flush with skull surface (0), or elevated, forming ridged orbital margins (1).

Character 267 (Brochu, 1997: char. 130): Capitate process of laterosphenoid oriented laterally (0) or anteroposteriorly (1) toward midline.

Character 268 (modified from Brochu, 1997: char. 141): Paroccipital process development lateral to cranioquadrate opening: short (0) or long (1).

Character 269 (modified from Norell, 1988: char. 32 by Brochu, 1997: char. 149): Ectopterygoid extends (0) or does not extend (1) to posterior tip of lateral pterygoid flange at maturity.

Character 270 (Brochu, 1997: char. 153): Incisive foramen completely situated far from premaxillary toothrow, at the level of the second or third alveolus (0) or abuts premaxillary toothrow (1).

Character 271 (Pritchard et al., 2012; modified from Pol et al., 2009 and Turner, 2004: character 126): Ventral surface of choanal septum smooth to slightly depressed (0), marked by an acute groove (1); or, vomeral choanal septum divided into bilateral laminae.

Character 272 (modified from Turner, 2006: char. 128): Proximal-most portion of fibular head straight sided to weakly developed posteriorly (0) or very sharply projecting posteriorly, forming distinct extension (1).

Character 273 (Turner, 2006: char. 129): Posterior process of cervical rib shaft lacks (0) or possesses (1) a posterodorsally projecting spine at the junction with the tubercular process.

Character 274 (Pol et al., 2009: char. 274): Longitudinal keels on dorsal surface of osteoderms restricted to the posterior edge of osteoderm (0) or are not (1).

Character 275 (Pol et al., 2009: char. 275): Jugal below the anteroventral corner of the orbit: lacks (0) or possesses an emarginated orbital margin and an associated depression located on the dorsal region of the jugal (1).

Character 276 (Pritchard et al., 2012; modified from Pol et al., 2009: char. 276): Transverse ridge crossing frontal anteromedial to orbits: absent (0), present as ridge (1), prominent anteriorly curved shelf (transverse interorbital crest sensu Andrade and Hornung, 2011) present (2), or anteroposteriorly oriented crest on frontal (3).

Character 277 (Pol et al., 2009: char. 277): Shallow hemispherical depression on the lacrimal and/or prefrontal anterior to the orbital margin (not articulation facet for palpebral): absent (0), or present (1).

Character 278 (Pol et al., 2009: char. 278): Anterior half of palatines between suborbital fenestrae: lateral margins are parallel to subparallel (0) or flared anteriorly (1).

Character 279 (modified from Pol et al., 2009: char. 279 and Montefeltro et al., 2011: char. 41): + Posterior half of palatines between suborbital fenestrae: lateral margins are parallel to subparallel (0) or slightly constricted and flared posteriorly (1), or markedly constricted lateromedially at its posterior portion and flaring posteriorly (2).

Character 280 (Pol et al., 2009: char. 280): Posteroventral margin of the angular straight or gently arched dorsally (0) or strongly arched dorsally (1).

Character 281 (Pol et al., 2009: char. 281): Lateral margin of dorsal surface of squamosal squared off with continuous ear valve groove (0), or bears a prominent depressed area just anterior to the posterior lobe of the squamosal, groove for ear valve discontinuous (1). The posterior end of the squamosal lobe as flares distally.

Character 282 (Pol et al., 2009: char. 282):Fibular shaft distal to iliofibularis trochanter straight (0) or bowed posteriorly (1).

Character 283 (Larsson and Sues, 2007: char. 55): Premaxillary teeth 1 and 2, position: separated like adjacent teeth (0), or nearly confluent (1).

Character 284 (Larsson and Sues, 2007: char. 60): Large nutrient foramen on palatal surface of premaxilla-maxilla contact: small or absent (0), or present (1).

Character 285 (Larsson and Sues, 2007: char. 62): Incisive foramen size: present and large (length equal or more than half the greatest width of premaxillae) (0), or present or small (1), or absent (2).

Character 286 (Larsson and Sues, 2007: char. 66): Premaxilla-maxilla lateral fossa excavating alveolous of last premaxillary tooth: no (0), or yes (1).

Character 287 (Pol and Powell, 2011: char. 287): Shape of antorbital fenestra: rounded or dorsoventrally high (0), or low and elongated, slit-like (1).

Character 288 (Pol and Powell, 2011: char. 288): Nasal exposure on lateral surface of rostrum: deflecting gradually from the dorsal surface (0), or deflecting abruptly, forming an almost 90 degree angle between the dorsal and lateral surfaces (1).

Character 289 (Pol and Powell, 2011: char. 289): Paired crests along the prefrontal-frontal sutures: absent (0), or present (1).

Character 290 (Pol and Powell, 2011: char. 290): Dorsal surface of frontal: flat or slightly concave (0), with a broad basin-like depressed area bordered posteriorly by a transversal ridge (1).

Character 291 (Pol and Powell, 2011: char. 291): Rugose surface on palatal surface of maxilla posterior to last tooth: absent (0), or present (1).

Character 292 (Pol and Powell, 2011: char. 292): Ectopterygoid-palatine contact posterior to the suborbital fenestra: not contacting (0), or contacting (1).

Character 293 (modified from Andrade and Bertini 2008a: char. 103 by Pol and Powell, 2011: char. 293): Pterygoid ventral surface at the origin of the pterygoid flanges: flat or slightly concave (0), or bearing a pterygoid parachoanal fossa located laterally or posterolaterally to choanal opening; a distinctly depressed area that perforates the pterygoid flanges in some taxa (1).

Character 294 (modified from Turner and Buckley, 2008: char. 286): Jugal, anterior and posterior processes: inline dorsoventrally (0) or dorsal margin of anterior and posterior processes at a sharp angle to one another, both processes slope ventrally to form a strongly arched jugal (1).

Character 295 (Larsson and Sues, 2007: char. 31): Length of anterior process of quadratojugal: either short or absent (0), or from long (less than half length of lower temporal bar) to moderate (one third of lower temporal bar) (1), or long (greater than half of lower temporal bar) (2).

Character 296 (Pol et al., 2012: char. 296). Prezygapophyseal process of anterior cervical vertebrae: anterodorsally projected and straight or slightly recurved (0), or dorsally projected and strongly recurved (1).

Character 297 (Pol et al., 2012: char. 297). Prezygapophyseal process of anterior to mid cervical vertebrae in lateral view: anterior margin straight or evenly convex (0), or anterior margin bearing a distinct bulge at the midpoint of the prezygapophyseal process (1).

Character 298 (Pol et al., 2012: char. 298). Shape of the articular surface of the parapophysis in posterior cervical and anterior dorsals: subcircular or ovoid with the major axis oriented anteroposteriorly (0), or subtriangular or ovoid with major axis oriented dorsoventrally (1).

Character 299 (modified from Pol et al., 2012: char. 299). Number of dorsal veretebrae with the parapophyses between the level of the base and the roof of the neural canal: one or less (0), or two or more (1).

Character 300 (Pol et al., 2012: char. 300). Medial surface of prezygapophyseal process of anterior to mid cervical vertebrae: with an ovoid or triangular depression close to the neural canal (0), or flat or slightly convex (1).

Character 301 (Pol et al., 2012: char. 301). Spinopostzygapophyseal lamina in dorsal vertebrae: absent (0), or present as a high and sharp lamina (1).

Character 302 (Pol et al., 2012: char. 302). Distinct rounded depression on the dorsal surface of neural arches of the anterior to mid dorsal vertebrae, located between the base of the neural spine and the postzygapophyseal process: absent (0), or present (1).

Character 303 (Pol et al., 2012: char. 303). Relative position of the transverse process and the postzygapophysis in mid dorsal vertebrae: postzygapophysis located dorsally to the transverse process (0), or postzygapophysis leveled with the transverse process (1).

Character 304 (Pol et al., 2012: char. 304). Dorsolateral end of first sacral rib: located at the level of the neural canal (0), or dorsoventrally expanded, projecting dorsally above the level of the neural canal (1).

Character 305 (Buckley and Brochu, 1999; char 106).Scapular blade no more than twice the length of the scapulocoracoid articulation (0), or scapular blade very broad and greater than twice the length of the scapulocoracoid articulation (1).

Character 306 (Pol et al., 2012: char. 306). Insertion mark dorsal to the glenoid facet of the scapula for the attachment of the M. triceps: present as a well-developed ridge or tubercle (0), or absent (1).

Character 307 (Pol et al., 2012: char. 307). Recess ventral to the glenoid facet of the coracoid: shallow and smoothly concave surface (0), or deep recess strongly concave in lateral view, overhung by a large ventral projection of the glenoid facet (1).

Character 308 (Pol et al., 2012: char. 308). Ventral expansion of the coracoid: larger or equal to the proximal expansion (0), or less expanded than the proximal region (1).

Character 309 (Pol et al., 2012: char. 309). Orientation of the area of instertion of M. subscapularis above the internal tuberosity of the humerus: obliquely oriented in anterior view, with the area of insertion facing proximomedially (0), or vertically oriented in anterior view, with the area of insertion facing medially (1).

Character 310 (Pol et al., 2012: char. 310). Anterior projection and profile of deltopectoral crest in humerus: Well-developed crest bearing a pointed tubercle for the insertion of the supracoracoideus complex (sensu Meers, 2003) (0), or low and anteriorly convex in lateral view, lacking a well-developed tubercle (1).

Character 311 (Pol et al., 2012: char. 311). Proximal third of the deltopectoral crest: originating at the proximolateral corner of the humerus and running distally along the proximal region of the lateral margin of the humerus (0), or proximal origin medially displaced from the proximolateral corner of the humerus and running distally, leaving an anteriorly facing concave surface between the crest and the lateral margin of the anterior surface of the humerus (which probably corresponds to the insertion area of the M. coracobrachialis brevis dorsalis) (1).

Character 312 (Pol et al., 2012: char. 312). Orientation and extension of the distal half of the deltopectoral crest: running along the lateral edge of the humerus or slightly deflected medially reaching, at the most, the lateromedial midpoint of the humeral shaft (0), or strongly deflected medially, surpassing the lateromedial midpoint of the anterior surface of the humeral shaft (1).

Character 313 (Pol et al., 2012: char. 313). Anterior surface of the distal half of the deltopectoral crest: lateromedially narrow, forming a sharp ridge, in some cases with a slightly bulged apex (0), or lateromedially broad forming an expanded anterior surface (1).

Character 314 (Pol et al., 2012: char. 314). Circular depression on the posterior surface of the proximal end of the humerus, related to the insertion of the M. scapulohumeralis caudalis: absent (0), or present (1).

Character 315 (Pol et al., 2012: char. 315). Posterior surface of the humerus with a distinct, sharply-delimited, pit at the proximodistal level of the apex of the deltopectoral crest, usually related to the insertion of the M. teres major and M. latissimus dorsi (sensu Meers, 2003): absent (0), or present (1).

Character 316 (Pol et al., 2012: char. 316). Anteroproximal end of the distal articular surface of the humerus: continuous with the anterior surface of the humeral shaft or incipiently projected anteriorly (0), or separated from the humeral shaft by a distinct step, formed by a concave and proximally facing shelf surface that extends lateromedially across the entire width of the distal humerus and is bound by two well developed supracondylar ridges (1).

Character 317 (Pol et al., 2012: char. 317). Lateral and medial surface of distal end of humerus: flat and anteroposteriorly broad, similar in anteroposterior length to the lateromedial width of the distal end of humerus (0), or convex and reduced in comparison with the lateromedial width of the distal humerus (1).

Character 318 (Pol et al., 2012: char. 318). Articular surface for the ulna on the radiale: facing posterolaterally (0), or facing posteriorly, not visible in lateral view (1).

Character 319 (Pol et al., 2012: char. 319). Proximodistal development of articular surface for the ulna on the radiale: short and wide, being up to than 30% of the total length of the radiale (0), or proximodistally elongated, being more than 40% of the total length of the radiale (1).

Character 320 (Pol et al., 2012: char. 320). Distal region of articular surface for the ulnare in the radiale: merging gradually with the posterolateral surface of the ulnar shaft (0), or usually triangular shaped, and separated from the ulnar shaft by a distinct step (1).

Character 321 (Pol et al., 2012: char. 321). Proximal region of articular surface for the ulnare in the radiale: divided from the articular surface for the ulna by a crest, creating a distinct articular surface for the ulnare (0), or continuous with the articular surface for the ulna (1).

Character 322 (Pol et al., 2012: char. 322). Anterior surface of radiale: smoothly convex (0), or bearing a proximodistal crest that extends along the shaft dividing the anterior surface of the radiale (1).

Character 323 (Pol et al., 2012: char. 323). Distolateral expansion of the ulnare: absent, as (or less) expanded as the distomedial corner of the ulnare (0), or distinctly expanded and projecting more distally than the distomedial corner of the ulnare, forming a distinct process (“ulnar anterior projection” sensu Nascimento and Zaher, 2010) (1).

Character 324 (Pol et al., 2012: char. 324). Lateromedial width of shaft of metacarpal I: as broad as the shaft of other metacarpals (0), or broader than other metacarpals, being the digit I the most robust element of the metacarpus (1).

Character 325 (Pol et al., 2012: char. 325). Development of the postacetabular process of the ilium: well developed as a distinct process that extends anteroposteriorly at least 60% of the acetabular length (0), or extremely reduced or absent, extending anteroposteriorly not more than 50% of the acetabular length (1).

Character 326 (Pol et al., 2012: char. 326). Posterior end of the postacetabular process: tapering posteriorly and ending in an acute tip (0), or subrectangular shaped with the posterior end vertically oriented, with its dorsoventral height being at least 60% of the height at the origin of the postacetabular process (1).

Character 327 (Pol et al., 2012: char. 327). Orientation of the ventral margin of the postacetabular process: posterodorsally directed (0), or horizontally or slightly posteroventrally deflected (1).

Character 328 (Pol et al., 2012: char. 328). Dorsoventral position of the ventral margin of the postacetabular process (along its posterior third): located at the same height or dorsally than the acetabular roof (0), or located at or ventrally than the dorsoventral midpoint of the acetabular height (1).

Character 329 (Pol et al., 2012: char. 329). Relative position of supraacetabular crest and iliac blade at the anterior region of the acetabulum of the ilium: well separated from each other by a shallow concave surface (0), merged together forming a single rugose surface for the insertion of the M. iliotibialis 1 and 2 (sensu Romer, 1923) (1).

Character 330 (Pol et al., 2012: char. 330). Anterior peduncle of ilium: shallow concavity separating the anterior and posterior articular surface of the anterior iliac peduncle (0), or deep notch incising two well developed articular surfaces, which project anteroventrally forming an acute angle between them (1).

Character 331 (Pol et al., 2012: char. 331). Development of greater trochanter on proximal femur: prominent, ridge-like lateral border that separates lateral surface of proximal femur from a flat posterior surface of proximal femur reaching down to the level of the fourth trochanter (0), or proximodistally short trochanteric surface lacking a distinct ridge that separates the lateral and posterior surfaces of the proximal femur and ending well above the fourth trochanter (1).

Character 332 (Pol et al., 2012: char. 332). Medial edge of the greater trochanter: low ridge or convex surface (0), or forms a prominent sharp long crest offset from the medial surface of the femur (1).

Character 333 (Pol et al., 2012: char. 333). Development of insertion scar for PIFI1 and CFL anterior to fourth trochanter: deep and rugose surface (0), or shallow and smooth depression (1).

Character 334 (Pol et al., 2012: char. 334). Lateral supracondylar ridge on anterior surface of distal femur: prominent and broad lateral suprancondylar ridge separating the anterior concave surface of femur from the lateral surface (0), or absence of well developed lateral suprancondylar ridge, anterior surface of femur flat or slightly concave and continuous with the lateral surface of the distal femur (1).

Character 335 (Pol et al., 2012: char. 335). Distal half of tibial shaft in lateral view: straight (0), or posteriorly bowed (1).

Character 336 (Pol et al., 2012: char. 336). Tibial shaft in anterior or posterior view: straight or only slightly bowed (0), or markedly bowed laterally (1).

Character 337 (Pol et al., 2012: char. 337). Distal projection of tibial articular surfaces: medial region of distal articular surface of distal tibia extends further distally than the lateral region, forming a strongly oblique distal margin of the tibia (0), or medial and lateral regions subequally extended, with distal margin subhorizontally oriented (1).

Character 338 (Pol et al., 2012: char. 338). Anterior margin of the tibial facet on the astragalus: forming a well-defined ridge that reaches medially the ball-shaped region for the articulation of metatarsal I-II and closes the proximomedial corner of the anterior hollow of the astragalus (0), or forming a low ridge that is medially separated by a notch from the ball-shaped region for the articulation of the metatarsals I-II, failing to close the proximomedial corner of the anterior hollow (1).

Character 339 (Pol et al., 2012: char. 339). Planar and proximal calcaneal surfaces on the astragalus: connected to each other forming a continuous articular surface that articulates with the calcaneal condyle, the margin of which forms the distolateral ridge-like margin of the anterior hollow of the astragalus (0), or separated from each other forming two distinct articular surfaces for the planar and proximal articular surfaces of the calcaneum (1).

Character 340 (Pol et al., 2012: char. 340). Articular surface for the distal tarsal 3 on astragalus: proximodistally leveled with the distal end of the planar calcaneal facet and distal surface of the ball-like articulation for metatarsals I-II, in anterior view these structures form an elevated ridge that close the distal corner of the anterior hollow of the astagalus (0), or proximally inset creating a clear separation between the planar facet and the distal surface of the ball like articulation for metatarsals I-II, and leaving a distal notch along the margins of the anterior astragalar hollow (1).

Character 341 (modified from Pol et al., 2012: char. 341). Astragalar-tarsal ligament pit on astragalus (sensu Sertich and Groenke, 2010) at the distal end of the anterior hollow: not differentiated from the rest of the anterior hollow of the astragalus (0), or distinct depression (1).

Character 342 (Pol et al., 2012: char. 342). Development of proximal astragalar depression, located posteriorly to the tibial facet of the astragalus: shallow concave depression (0), or deep depression with sharply delimited medial and anterior margins, forming a true astragalar fossa (1).

Character 343 (Pol et al., 2012: char. 343). Shape of the fibular facet on the astragalus: + subtrapezoidal with the proximodistal height of anterior margin higher than the posterior margin (0), or subrectangular with subequal anterior and posterior margins (1), or trapezoidal with the proximodistal height of its anterior margin lower that the posterior margin (2).

Character 344 (Pol et al., 2012: char. 344). Ridge along dorsolateral edge of calcaneal tuber and associated fossa medially to the ridge: present (0), or absent (1).

Character 345 (Pol et al., 2012: char. 345). Calcaneal tuber with lateral tubercle and crest extending anteriorly from it: present (0), or absent (1).

Character 346 (Pol et al., 2012: char. 346). Posterolateral region of the facet for distal tarsal 4 in calcaneum: subrectangular with a right-angled posterolateral corner (0), or subtriangular shaped with an oblique posterolateral margin (1).

Character 347 (Pol et al., 2012: char. 347). Calcaneum with posterior astragalar facet: subtriangular with proximal and lateral margins forming a right angle and an oblique medioplantar edge (0), or proximal and plantar edges subparallel to each other connected through a broad and rounded medial margin (1).

Character 348 (modified from Novas et al., 2009: char. 231): Anterior margin of the suborbital fenestra: maxilla precludes the ectopterygoid-palatine contact at the anterior margin of the suborbital fenestra (0), or ectopterygoid prjects anteromedially contacting (or almost reaching) the anterolateral end of the palatine, mostly or completely excluding the maxilla from the anterior margin of the suborbital fenestra (1).

Character 349 (modified from Novas et al., 2009: char. 232): Posterior end of the glenoid facet of articular: located above the surangular-angular suture (0), or ventrally recessed, located at or below the dorsoventral midpoint of the posterior mandibular ramus (i.e., surangular forming a high lateral wall that covers the posterior end of the glenoid facet) (1).

Character 350 (Novas et al., 2009: char. 233): Ventral margin of the lateral edge of squamosal, above otic recess: straight or slightly sinusoidal (0) or bearing a highly convex ventral outgrowth anteriorly to a small but highly concave concavity located at the level of the otic aperture (1).

Character 351 (Pol et al., in press): Jugal anteroventral process between maxilla and ectopterygoid: absent (0), present, jugal extending anteriorly a short triangular process that wedges between the ecotpterygoid and maxilla on the lateroventral surface of the skull at the level of the orbits (“sickle-like medial process present on the ventral surface of the anterior jugal ramus” sensu Andrade and Bertini, 2008a) (1).

Character 352 (Pol et al., in press): Posterior maxillary surface at the anteroventral region of the orbit: dorsoventrally thin and horizontal, forming the posterior end of the palatal branch (0), or forming an orbital lamina, a vertical wall that restricts the opening of the nasal cavity into the orbit (1).

Character 353 (Pol et al., in press): Frontal shape along its suture with the prefrontal: relatively broad and tapering gradually anteriorly (0), or broad tabular-shaped with lateral sutures with prefrontals parallel to each other (1).

Character 354 (Pol et al., in press): Temporo-orbital foramen: enclosed between the parietal and squamosal (0), or completely enclosed within squamosal (1).

Character 355 (Pol et al., in press): Ornamentation on dorsal surface of the posterolateral process of squamosal: present (0), or absent (1).

Character 356 (modified from Sereno and Larsson, 2009: char. 69): + Anterior extension of the otic recess: restricted to the squamosal (0), or extends on the posterior region of the lateral surface of the postorbital (1), or extends along the entire length of the postorbital, which has an anterior transverse lamina that separates the otic recess from the orbit (2).

Character 357 (modified from Andrade and Bertini 2008a: char. 70): + Quadrate contact with basioccipital: absent (0), or located on the ventral surface of the braincase (1), or well developed medial crest of quadrate meets the basioccipital on the occipital surface of the skull, excluding the exoccipital from the ventral margin of the occipital surface (2).

Character 358 (Pol et al., in press): Supraoccipital lateromedial width: extensive, occupying half of the lateromedial width of the occipital table (0), or narrow, occupying less than one third of the lateromedial width of the occipital table (1).

Character 359 (Pol et al., in press): Entrance of internal carotid artery into occipital surface of the skull: located close to the ventral end of the exoccipital, ventrally separated from the opening for the cranial nerves IX-XI (0), located dorsally, close to and within the same depression as the foramina for the cranial nerves IX-XI (1).

Character 360 (Turner and Sertich, 2010: char 297): Sagittal ridge on the ventral half of the posterior surface of the basioccipital: absent or poorly developed (0), or present (1).

Character 361 (Pol et al., in press): Palatine width at the level of the anterior end of suborbital fenestra: broad, close to half the width of the maxillary palate (0), or narrow, approximately 25% the withd of the maxillary palate (1).

Character 362 (modified from Montefeltro et al. 2011: char. 44): Longitudinal sulcus (and associated foramina) on the ventral surface of palatines between suborbital fenestra: absent (0), or present (1).

Character 363 (Pol et al., in press): Anterior region of dentary symphysis in ventral view: lacking a distinct anterior process, lateral margin of the dentaries diverge gradually (0), or having a distinct anterior process with parallel lateral margins (1).

Character 364 (Pol et al., in press): + Relative length and width of anterior (parallel sided) process of dentary symphysis: short and as broad as long (0), elongated, being approximately twice as long as wide (1), or extremely long and narrow, being approximately three times as long as wide (2).

Character 365: Size of neurovascular foramina on mid to posterior region of alveolar edge of the dentary: small (0), or extremely large, being approximately as anteroposteirorly long as an alveolus (1).

Character 366 (Pol et al., in press): Sutural contact between dentary and surangular above the external mandibular fenestra: dentary overlaps surangular (0), or surangular overlaps dentary (1), or interdigitated and vertically oriented suture (2).

Character 367 (Modified from Andrade and Bertini, 2008a: char 113; Turner and Buckley, 2008: char 289): Posterodorsal branch of dentaries (above external mandibular fenestra): single branch sutured to the ventral margin of the anterior process of the surangular (0), divided into a ventral and a dorsal process exposed on the lateral surface of the lower jaw, the dorsal process fits into the large notch between the medial and lateral rami of the bifurcated anterior end of the surangular (1).

Character 368 (modified from Brochu, 1999: character 41) +: Location of the anterior opening for the mandibular nerve (V3): located at or close to the rostral margin of the splenial (0), or enclosed in the splenial and located on the anterior region of splenial (i.e., anterior foramen intermandibularis oralis sensu Brochu, 1999) (1), or enclosed in the splenial but located at the anteroposterior midpoint of the splenial (2).

Character 369 (Pol et al., in press): Foramen intermandibularis caudalis: present and enclosed between the angular and splenial below the mandibular adductor fossa (0), or absent with imperforated splenial-angular suture (1)

Character 370 (Pol et al., in press): Location of the posterior peg in mandibular symphysis: located on the ventral surface of symphysis (0), or located above the ventral surface, on the posterior surface of the symphysis (1)

Character 371 (Pol et al., in press): Smooth elongated fossa extending along ventral margin of external mandibular fenestra on the angular: absent, lateral surface of the angular reaching the ventral edge of the fenestra (0), or present, separated from the lateral surface of the angular by a sharp ridge (1).

Character 372 (Pol et al., in press): Coronoid tuberosities on the medial surface of anterior region of surangular: absent or poorly developed (0), well developed, forming prominent elongated crests divided by a deep longitudinal sulcus (1).

Character 373 (Pol et al., in press): Dorsal surfaces of the lateral glenoid facet and the lateral flange of the retroarticular process: glenoid facet separated from the retroarticular surface by a ridge or a step (0), or continuous (1).

Character 374 (Pol et al., in press): Length of the lateral flange of the retroarticular process relative to the lateromedial width of the glenoid facets of the articular: shorter (0), or approximately the same length or longer (1).

Character 375 (Pol et al., in press): Rounded bulge at the posterior end of the lateral flange of the retroarticular process: absent (0), or present (1).

Character 376 (Pol et al., in press): Orientation of the ridge on the dorsal surface of retroarticular process that divides the of the lateral and medial flanges of the retroarticular process: directed posteriorly, parallel to the longitudinal axis of the mandibular ramus (0), or directed posterolaterally, approximately at 45 degrees with the longitudinal axis of the mandibular ramus (1).

Character 377 (Pol et al., in press): Small bulge located proximally on the medial flange of the retroarticular process, posteriorly to the medial glenoid facet of the articular and associated with the foramen aerum in some taxa: absent (0), or present (1).

Character 378 (Pol et al., in press): Anteromedial end of medial flange of the retroarticular process: connected to the posteromedial corner of the medial glenoid facet of the articular through a dorsally directed crest (0), or extending anteriorly as a distinct anterior process up to the level of the anteroposterior midpoint of the medial glenoid of the articular (1), or projecting anteroventrally as deep pendant process (2).

Character 379 (Pol et al., in press): Orientation of medial flange of the retroarticular process: facing dorsally or slightly dorsomedially, having a similar orientation to the lateral flange to the medial flange of the retroarticular process (0), or facing medially, strongly deflected and forming an angle of approximately 90 degrees with the dorsal surface of the lateral flange (1).

Character 380 (Pol et al., in press): Medial edge of the medial flange of the retroarticular process: straight or slightly convex (0), or strongly convex forming a paddle-shaped medial flange; its margin forms an extensive arch of approximately half circumference when viewed in dorsal view (1).

Character 381 (modified from Andrade and Bertini 2008a: char. 128 and Turner and Sertich, 2010: char. 296): Transitional tooth located at the contact between the premaxilla and maxilla, both of which contribute to the alveolar walls: absent (0), or present (1).

Character 382 (Pol et al., in press): Number of strongly procumbent teeth on the anterior region the mandibular symphysis: one tooth on each dentary (0), or two procumbent teeth on each dentary (1).

Character 383 (Pol et al., in press): Implantation of lower incisiviforms: in separate alveoli (0), or in a continuous alveolar groove (1).

Character 384 (modified from Andrade et al., 2011: char. 399): Left and right toothrow along mandibular symphysis: well separated from each other by a broad dorsal surface of the symphysis (0), or closely located to each other (forming a symphyseal tooth battery in most taxa) (1).

Character 385 (Pol et al., in press): Apico-basal ridges on the enamel surface of incisiviforms and caniniform: absent (0), or well-developed (1).

Character 386 (modified from Andrade and Bertini 2008a: char. 123): Apico-basal ridges on the enamel surface of posterior teeth: absent (0), or present (1).

Character 387 (Pol et al., in press): Separation of apico-basal ridges on the enamel surface of teeth: fine enamel ridges that are closely spaced to each other (flutting) (0), or ridges, usually with a broad base, well spaced from each other (1).

Character 388 (Pol et al., in press): Size variation of denticles along denticulated carinae: absent or minor variation (0), or variable, with denticles at the central region of the carinae being approximately twice the size (height and width) of both apical and basal denticles (1), or decreasing gradually along the carina from the apex to the base of the crown, apical denticles are more than three times the height of the basal denticles (2)

Character 389 (Pol et al., in press): Thin enamel ridge (loph) connecting adjacent denticles instead of presenting distinct interdenticular slits: absent (0), or present (1)

Character 390 (modified from Andrade and Bertini, 2008a: char. 149 and O´Connor et al., 2010: char. 233): Horizontal cingula along the buccal and/or lingual margin of the base of the crown of postincisiform teeth: absent (0), or present, with accessory cusps and styli (1).

Character 391 (modified from Riff and Kellner, 2011: char 264): Posterior teeth with accessory apicobasally oriented keels bearing cusps or tuberous denticles located lingually and buccally from the major central keel: absent (0), present (1)

Character 392 (modified from Turner and Sertich, 2010: char. 294): Outer enamel surface (between carinae, apicobasal ridges, or flutting, if present): smooth (0), rugose (1)

Character 393 (modified from Andrade et al. 2011: char. 374): Rugose texture on outer enamel surface: formed by anastomizing grooves and ridges (0), formed by small globular protuberances (“pebbled enamel” sensu Price, 1950) closely spaced to each other (1)

Character 394 (modified from Andrade and Bertini 2008a: char. 138 and O´Connor et al. 2010: char 235): Tooth-tooth occlusion wear facets in posterior teeth: absent (0), present (1)

Character 395 (Pol et al., in press): Location and orientation of tooth-tooth occlusion wear facets in posterior teeth: oriented horizontally on the occlusal surface of the crown, parallel to the longitudinal plane of the skull (0), or located mesiolingually from the apex of the crown in upper teeth and buccodistally from the apex in lower teeth (Pol 2003: fig. 3; Lecuona and Pol 2008: fig. 1), oriented along a plane that is oblique to the longitudinal and sagittal planes of the skull (1)

Character 396 (Turner and Buckley, 2008: char. 290): Prominent depression on palate near alveolar margin at level of sixth or seventh alveolus: absent (0), or present (1).

Character 397 (modified from Turner and Sertich, 2010: char. 293): Gap on line of large neurovascular foramina on lateral surface of maxilla, along alveolar margin: absent, foramina form single continuous row (0), or present with a gap between anterior series and posterior series of foramina (1).

Character 398 (modified from Sereno and Larsson, 2009: char. 46): Lateral surface of jugal-ectopterygoid contact: inset from lateral jugal margin (0), or confluent with lateral jugal margin (1).

Character 399 (modified from Montefeltro et al., 2011: char. 45): Ventral margin of jugal at posterior end of ectopterygoid contact: continuous with the infratemporal bar of jugal (0), or suborbital region of jugal separated by a notch from infratemporal bar of jugal (1).

Character 400 (Sereno and Larsson, 2009: char. 83): Single or paired large neurovascular foramina on lateral surface of premaxilla, at its posterolateral corner: absent (0), or present (1).

Character 401 (modified from Montefeltro et al. 2011: char. 5): + Prefrontal-prefrontal medial contact: absent (with a broad contact between nasal and frontal) (0) anterior region of prefrontals project a medial pointed process that almost touch the other prefrontal (with a tiny contact between nasal and frontal) or touch each other as a punctual contact (1), contact present along mostly of the dorsal medial edge (2).

Character 402 (Montefeltro et al. 2011: char. 23): Maxillary palatal sagittal contact: smooth (0), bearing a longitudinal series of foramina (1).

Character 403 (modified from Montefeltro et al. 2011: char. 33): absent (0), present and elongated, reaching close to or extending into the quadratojugal-quadrate suture (1).

Character 404 (Montefeltro et al. 2011: char 34): Periotic quadrate fenestrae on lateral surface of quadrate: visible in lateral view (0), internalized in otic notch (1).

Character 405 (modified from Montefeltro et al 2011: char. 42): Medial palatal contact between suborbital fenestra: (0), distinctly raised forming a ridged suture along its whole extension (1).

Character 406 (Montefeltro et al., 2011: char. 64): Posteroventral symphyseal depressions: absent (0), present (1).

Character 407 (modified from Montefeltro et al., 2011: char. 11): Extension of frontal sagittal ridge: extending along the entire frontal dorsal surface (0), failing to reach the anterior end of the frontal, extending up to 75% of its anteroposteriorlength (1).

Character 408 (modified from Montefeltro et al., 2011: char. 37): Supraoccipital dorsal exposure on skull roof: subtriangular or crescentic shaped with the maximum anteroposterior length located along the sagittal plane and lateral regions anteroposteriorly shorter (0), forming a anteroposteriorly short but lateromedially broad surface sutured to the posteriormost portion of parietal and squamosal, with the lateral ends as anteroposteriorly long as the central region (1).

Character 409 (Larsson and Sues, 2007: char. 71): Sagittal torus on maxillary palatal shelves: absent (0), or present (1).

Character 410 (Pol et al., in press): Groove located on premaxillary lateral surface, running anteroventrally from the dorsoventral midpoint of its posterior margin: absent (0), or present (1).

Character 411 (modified from Nascimento and Zaher 2011: char. 258 and Montefeltro et al. 2011: char. 16): Suture between the postorbital and the squamosal in lateral view: straight or almost straight, vertical or oblique (0), or convex anteriorly (1).

Character 412 (Pol et al., in press): Anterolateral corner of supratemporal fossa: with a continuous rim formed by the postorbital dorsal surface (0), or with a transversely oriented groove on dorsal surface of postorbital interrupting the anterolateral rim of the supratemporal fossa (1).

Character 413 (Leardi et al., 2015b): Acromial region of the scapula: not distinctively expanded, forming an angle of more than 120º with the major axis of the scapula (0); or, expanded, with a cuadrangular proximal end in lateral view and forming an angle of 105º or less with the major axis of the scapula (1).

Character 414 (Leardi et al., 2015b): Glenoid facet on the scapula: Proximolaterally exposed (0); or, exposed proximally, being concave on lateral view, forming a dorsal roof to the glenoid facet of the coracoid (1).

Character 415 (Leardi et al., 2015b): Bulge on the anterior margin of the glenoid facet on the scapula: absent (0); or, present (1).

Character 416 (Leardi et al., 2015b): Posterior edge of the scapular blade: smooth (0); or, with a posterior flange that can be inferred as the insertion of the scapulosternal ligament (1).

Character 417 (Leardi et al., 2015b): Anteroposterior development of the glenoid facet on the coracoid: short, well posterior to the level of the coracoid foramen (0); or, long, reaching or almost reaching the level of the coracoid foramen (1).

Character 418 (Leardi et al., 2015b): Crest on the posterior surface of the proximal end of the humerus: passing just anteriorly to the scar of the common insertion of the M. teres major and M. latissimus dorsi (0); or, finishing dorsally to this scar (1).

Character 419 (Leardi et al., 2015b): Anterolateral process of the ulna: displaced anteriorly, leaving the proximal surface of articulation with the radius exposed anteriorly (0); or, on the lateral surface of the proximal end, leaving the proximal surface of articulation with the radius exposed anterolaterally (1).

Character 420 (Leardi et al., 2015b): Region between the anterolateral process of the ulna and the olecranon (or posterior process) in proximal view: convex (0); or, concave, bearing a well-developed furrow that extends onto the lateral surface of the ulna, that can be inferred as associated with the insertion of the M. flexor ulnaris (1).

Character 421 (Leardi et al., 2015b): Proximal (proximodorsal) process of the radiale: absent (0); or, present (1)

Character 422 (Leardi et al., 2015b): Ungual manual phalanges: lateromedially broad, with a convex and gently rounded ventral surface (0); or, lateromedially compressed, with a longitudinal crest along the whole ventral surface (1).

Character 423 (Leardi et al., 2015b): Anteroproximal process of the ischium: anteroposteriorly long and dorsoventrally low, with the distal end being 3 times smaller than the whole length of the process (0); or, anteroposteriorly short and dorsoventrally tall, with the distal end equal or almost equal to the whole length of the process (1).

Character 424 (Leardi et al., 2015b): Fibular facet on the fibular condyle of the femur: starting anteriorly to the level of the posterior end of the medial condyle (0); or, starting posteriorly to the level of the posterior end of the medial condyle (1).

Character 425 (Leardi et al., 2015b): Distal end of the fibula in distal view: rounded (0); or, triangular (1).

Character 426 (Leardi et al., 2015b): Strong dorsal crest separating the dorsal region of the anterior hollow of the astragalus from the astragalar-tarsale ligament pit: absent (0); or, present (1).

Character 427 (Leardi et al., 2015b): Vascular foramen on the anterior hollow: absent (0); present (1).

Character 428 (Leardi et al., 2015b): Strong median crest oriented proximodistally on the posterior surface of the astragalus connecting the distal roller with the posteromedial edge of the proximolateral process of the astragalus, closing posteriorly the proximal depression of the astragalus: absent or not continuous (0); or, present (1).

Character 429 (Leardi et al., 2015b): Dorsal surface of the calcaneal tuber: lacks (0); or, has a small nutrient foramen around its mid length (1).

Character 430 (Leardi et al., 2015b): Lateral bulge on the atlantal neural arch: absent (0); or, present (1).

Character 431 (Leardi et al., 2015b): Hypapophysis on the axis: absent or poorly developed (0); or, present as a strong anteroventral process (1).

Character 432 (Leardi et al., 2015b): Prespinal fossa on the cervical vertebrae: absent or shallow (0); or, deep, forming a strong depression on the dorsal surface of the neural arch (1).

Character 433 (Leardi et al., 2015b): Neural spines of the anterior cervical vertebrae: vertical or posteriorly slanted (0); or, anteriorly slanted (1).

Character 434 (Leardi et al., 2015b): Ventral keel on the ventral surface of the cervical vertebrae: absent or weak, not reaching the posterior margin of the centra (0); or, strongly developed, reaching the posterior margin of the centra (1).

Character 435 (Leardi et al., 2015b): Ventral keel on the ventral surface of the anterior dorsal vertebrae: absent or weak, not reaching the posterior margin of the centra (0); or, strongly developed, reaching the posterior margin of the centra (1).

Character 436 (Leardi et al., 2015b): Parapodiapophyseal laminae on the anterior dorsal vertebrae: absent or weakly developed (0); or, present as sharp laminae uniting the para and diapophyses, not leaving a concavity between both processes (1).

Character 437 (Leardi et al., 2015b): Last dorsal vertebrae in which the parapophyses are in contact with the neurocentral suture: D2 or anteriorly (0); D3 (1); or, D4 or posteriorly (2).

Character 438 (Leardi et al., 2015a): Sacral rib scars: Contacting each other (0); or, separated each other by a non-articular surface (1).

Character 439 (Fiorelli and Calvo, 2007): Ratio of femoral length versus tibial length: greater than one (femur longer than the tibia) (0); or, equal or smaller than 1 (tibia as long or longer than the femur).

**2.3 – Results of the phylogenetic analysis**

The strict consensus of the 1298 MPT’s with 1693 steps is depicted below.


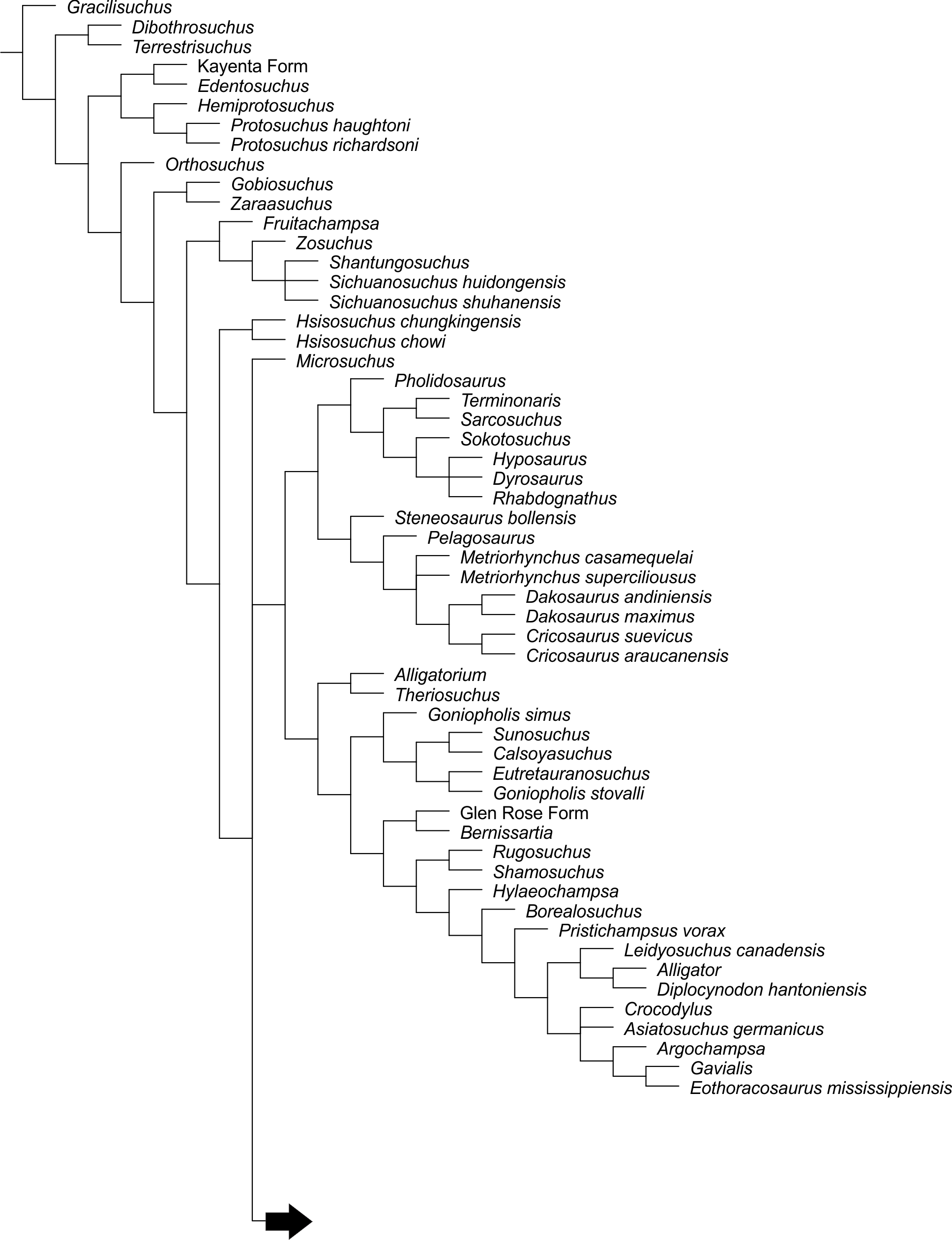


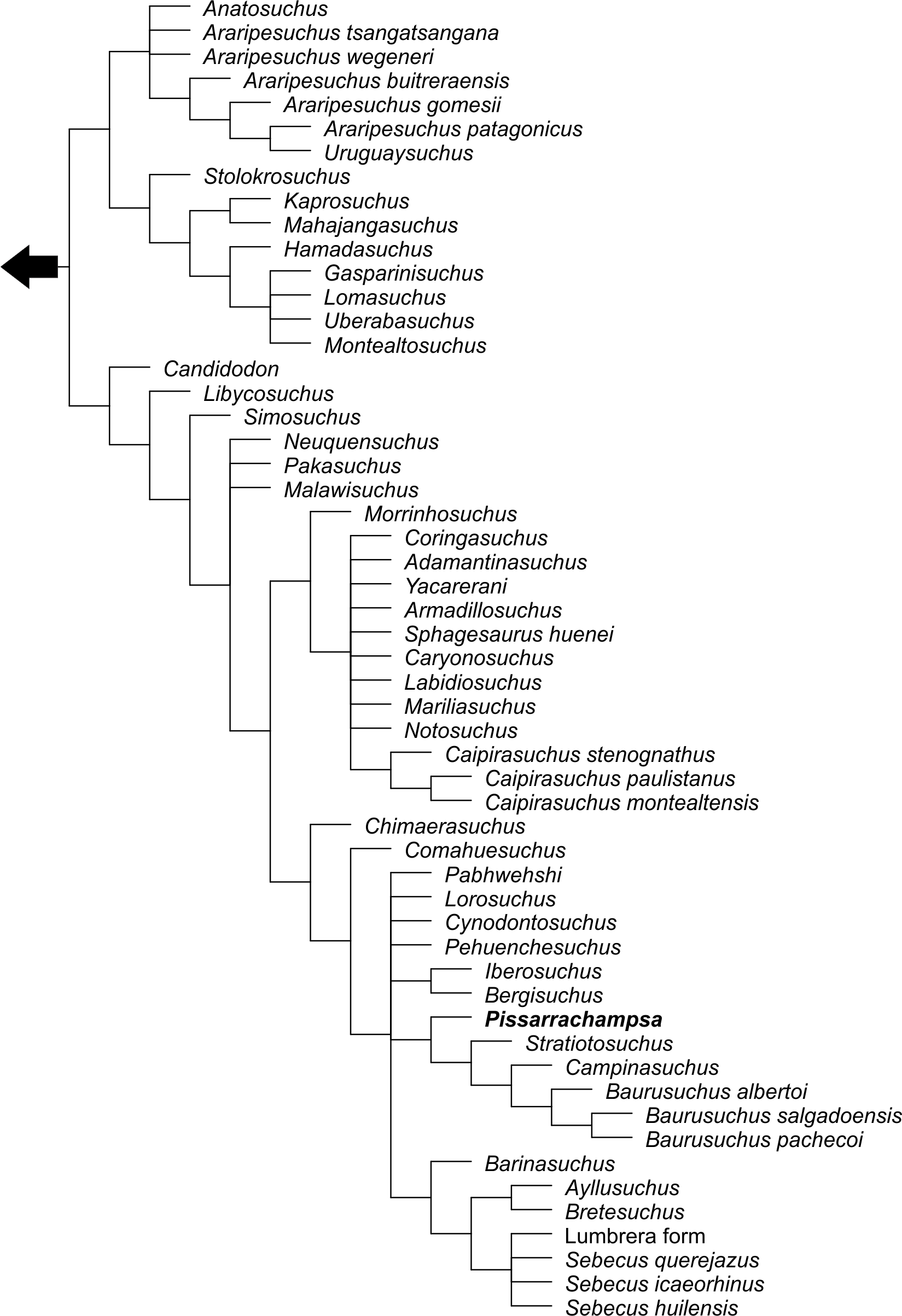


**Figure S1: Strict consensus of the 1298 MPT’s found in the analysis using the extended dataset based on Leardi *et al*. (2015a).**

**2.4 – Exploratory analyses**

As discussed in the main text, we conducted exploratory analyses in order to investigate the significance and influence of the postcranium in the results of the phylogenetic analysis we performed.

List of cranial characters

1-81, 102-108, 111-112, 118-150, 154-156, 158-176, 178-204, 206-220, 224-254, 256-257, 262-271, 275-281, 283-295, 348-412.

List of postcranial characters

82-101, 109-110, 113-117, 151-153, 157, 177, 205, 221-223, 255, 258-261, 272-274, 282, 296-347, 413-439.

Taxa pruning

The OTUs lacking postcranium or cranial information that were excluded from the matrix for the exploratory analyses are: *Zosuchus davidsoni, Libycosuchus brevirostris, Comahuesuchus brachybuccalis, Labidiosuchus amicum, Caryonosuchus pricei, Caipirasuchus stenognathus, Caipirasuchus montealtensis, Caipirasuchus paulistanus, Adamantinasuchus navae, Coringasuchus anisodontis, Morrinhosuchus luziae, Campinasuchus dinizi, Baurusuchus pachecoi, Pehuenchesuchus enderi, Cynodontosuchus rothi, Bergisuchus dietrichbergi, Bretesuchus bonapartei, Barinasuchus arveloi, Sebecus huillensis, Sebecus querejazus, Ayllusuchus fernandezi, Lorosuchus nodosus,* Lumbrera Form, *Pabweshi pakistanensis, Araripesuchus buitreraensis, Gasparinisuchus peirosauroides, Hamadasuchus rebouli, Kaprosuchus saharicus, Stolokrosuchus lapparenti, Hylaeochampsa vectiana, Metriorhynchus casamiquelai, Rhabdognathus, Sokotosuchus ianwilsoni, Argochampsa krebsi, Microsuchus schilleri,* and, *Neuquensuchus universitas. Candidodon itapecuruense, Calsoyasuchus valliceps,* and Glen Rose Form were excluded because a single character is scored among postcranial characters.

Strict consensus tree of the analysis for the dataset based on postcranial characters

The strict consensus of the 9 MPT’s recovered with 280 steps is depicted below.


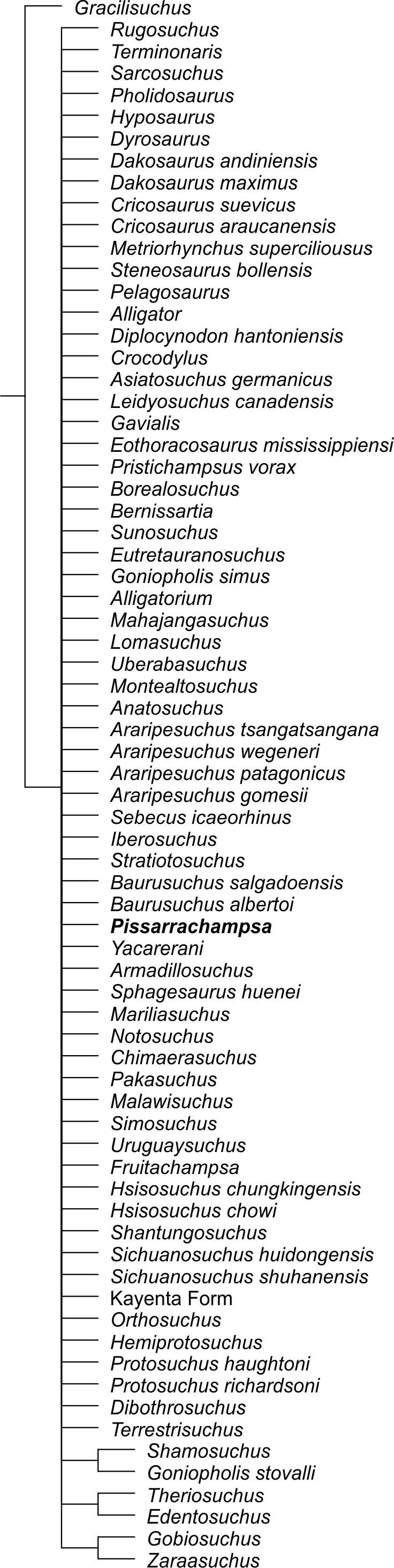


**Figure S2: Strict consensus of the 9 MPT’s found in the analysis using only postcranial characters.**

**3 – Supplementary literature cited**

Andrade M. B., and Bertini R. J. 2008a. A new *Sphagesaurus* (Mesoeucrocodylia: Notosuchia) from the Upper Cretaceous of Monte Alto City (Bauru Group, Brazil), and a revision of the Sphagesauridae. *Historical Biology* 20:101–136.

Andrade M. B., and Bertini R. J. 2008b. Morphology of the dental carinae in *Mariliasuchus amarali* (Crocodylomorpha, Notosuchia) and the pattern of tooth serration among basal mesoeucrocodylia. *Arquivos do Museu Nacional* 66:63–82.

Andrade M. B., Edmonds, R., Benton, M. J., and Schouten, R. 2011. A new Berriasian species of *Goniopholis* (Mesoeucrocodylia, Neosuchia) from England, and a review of the genus. *Zoological Journal of the Linnean Society* 163:S66–S108.

Brochu, C. A. 1997. Fossils, morphology, divergence timing, and the phylogenetic relationships of *Gavialis*. *Systematic Biology* 46:479–522.

Brochu, C. A. 1999. Phylogenetics, taxonomy, and historical biogeography of Alligatoroidea. *Memoir of the Society of Vertebrate Paleontology* 4:1–92.

Buckley, G. A. and Brochu, C.A. 1999. An enigmatic new crocodile from the Upper Cretaceous of Madagascar. *Special Papers in Palaeontology* 60:149–175.

Buscalioni, A. D., and Sanz, J. L. 1988. Phylogenetic relationships of the Atoposauridae (Archosauria, Crocodylomorpha). *Historical Biology* 1:233–250.

Campione N. E., and Evans D. C. 2012. A universal scaling relationship between body mass and proximal limb bone dimensions in quadrupedal terrestrial tetrapods. *BMC Biology*, 10: 1–21.Clark, J. M. 1994. Patterns of evolution in Mesozoic Crocodyliformes. In: N. C. Fraser and H.-D. Sues (eds.) *In the shadow of dinosaurs*. Cambridge University Press, Cambridge, pp. 84–97.

Farlow J. O., Hurlburt G. R., Elsey R. M., Britton A. R., and Langston Jr, W. 2005. Femoral dimensions and body size of *Alligator mississippiensis*: estimating the size of extinct mesoeucrocodylians*. Journal of Vertebrate Paleontology* 25:354–369.

Fiorelli, L. E and Calvo, J. O. 2007. The first “protosuchian” (Archosauria: Crocodyliformes) from the Cretaceous (Santonian) of Gondwana. *Arquivos do Museu Nacional* 65:417–459.

Godoy, P. L, Montefeltro F. C, Norell M. A, and Langer M. C. 2014. An additional baurusuchid from the Cretaceous of Brazil with evidence of interspecific predation among Crocodyliformes. PLoS ONE 9 e97138.

Gomani, E. M. 1997.A crocodyliform from the Early Cretaceous Dinosaur Beds, Northern Malawi. *Journal of Vertebrate Paleontology* 17:280–294.

Larsson, H. C. E., and Sues, H.-D. 2007. Cranial osteology and phylogenetic relationships of *Hamadasuchus rebouli* (Crocodyliformes: Mesoeucrocodylia) from the Cretaceous of Morocco. *Zoological Journal of the Linnean Society* 149:533–567.

Leardi J. M., Fiorelli L. E., and Gasparini Z. 2015a. Redescription and reevaluation of the taxonomical status of *Microsuchus* *schilleri* (Crocodyliformes: Mesoeucrocodylia) from the Upper Cretaceous of Neuquén, Argentina. *Cretaceous Research* 52:153–166.

Leardi J. M., Pol D., Novas F. E., and Suarez Riglos M. 2015b. The postcranial anatomy of *Yacarerani* *boliviensis* and the phylogenetic significance of the notosuchian postcranial skeleton. *Journal of Vertebrate Paleontology* e995187.

Montefeltro, F. C., Larsson, H. C. E., and Langer, M. C. 2011.A new baurusuchid (Crocodyliformes, Mesoeucrocodylia) from the Late Cretaceous of Brazil and the phylogeny of Baurusuchidae. *PLoS ONE* 6: 1-26.

Nascimento P. M. 2008. Descrição morfológica e posicionamento filogenético de um Baurusuchidae (Crocodyliformes, Mesoeucrocodylia) do Cretáceo Superior da Bacia Bauru, região de General Salgado (SP). MSc Thesis, Universidade de São Paulo.

Nascimento, P. M., and Zaher, H. 2010. A new species of *Baurusuchus* (Crocodyliformes, Mesoeucrocodylia) from the Upper Cretaceous of Brazil, with the first complete postcranial skeleton described for the family Baurusuchidae. *Papéis Avulsos de Zoologia* 50:323–361.

Novas, F. E., Pais, D. F., Pol, D., Carvalho, I. S., Scanferla, A., Mones, A., and Suarez Riglos, M. 2009. Bizarre notosuchian crocodyliform with associated eggs from the Upper Cretaceous of Bolivia. *Journal of Vertebrate Paleontology* 29:1316-1320.

Ortega, F., Buscalioni, A. D. and Z. Gasparini. 1996. Reinterpretation and new denomination of *Atacisaurus crassiproratus* (Middle Eocene; Issel, France) as cf. *Iberosuchus* (Crocodylomorpha: Metasuchia). *Geobios* 29:353–364.

Ortega, F., Gasparini, Z., Buscalioni, A. D., and Calvo, J. O. 2000. A new species of *Araripesuchus* (Crocodylomorpha, Mesoeucrocodylia) from the Lower Cretaceous of Patagonia (Argentina). *Journal of Vertebrate Paleontology* 20:57–76.

Pol, D. 1999a. El esqueleto postcraneano de *Notosuchus terrestris* (Archosauria: Crocodyliformes) del Cretácico Superior de la Cuenca Neuquina y su información filogenética. PhD (Licenciatura) Thesis, Facultad de Ciencias Exactas y Naturales, Universidad de Buenos Aires, 158 pp.

Pol, D.1999b. Basal mesoeucrocodylian relationships: new clues to old conflicts. *Journal of Vertebrate Paleontology* 19:69A.

Pol, D. 2003.New remains of *Sphagesaurus huenei* (Crocodylomorpha: Mesoeucrocodylia) from the Late Cretaceous of Brazil. *Journal of Vertebrate Paleontology* 23:817–831.

Pol, D., and Apesteguía, S. 2005. New *Araripesuchus* remains from the Early Late Cretaceous (Cenomanian) of Patagonia. *American Museum Novitates* 3490:1–38.

Pol, D., and Gasparini, Z. 2009. Skull Anatomy of *Dakosaurus andiniensis* (Thalattosuchia: Crocodylomorpha) and the phylogentic position of Thalattosuchia. *Journal of Systematic Palaeontology* 7:165–197.

Pol, D., and Norell, M. A. 2004a. A new crocodyliform from Zos Canyon Mongolia. *American Museum Novitates* 3445:1–36.

Pol, D., and Norell, M. A. 2004b. A new gobiosuchid crocodyliform taxon from the Cretaceous of Mongolia. *American Museum Novitates* 3458:1–31.

Pol, D., and Powell, J. E. 2011. A new basal mesoeucrocodylian from the Río Loro Formation (Paleocene) of northwestern Argentina. *Zoological Journal of the Linnean Society* 163:S7–S36.

Pol, D., Ji, S.-H., Clark, J. M., and Chiappe, L. M. 2004. Basal crocodyliforms from the Early Cretaceous Tugulu Group (Xinjiang, China), and the phylogeneticposition of *Edentosuchus*. *Cretaceous Research* 25:603–622.

Pol, D., Turner, A. H., and Norell, M. A. 2009. Morphology of the Late Cretaceous crocodylomorph *Shamosuchus djadochtaensis* and a discussion of neosuchian phylogeny as related to the origin of Eusuchia. *Bulletin of American Museum of Natural History* 324:1–103.

Pol D., Leardi J. M., Lecuona A., and Krause M. 2012. Postcranial anatomy of *Sebecus icaeorhinus* (Crocodyliformes, Sebecidae) from the Eocene of Patagonia. *Journal of Vertebrate Paleontology* 32:328–354.

Pol D., Nascimento P. M., Carvalho A. B., Riccomini C., Pires-Domingues R. A., and Zaher H. 2014. A new notosuchian from the Late Cretaceous of Brazil and the phylogeny of advanced notosuchians. *PLoS ONE* 9 e93105.

Pritchard, A. C., Turner, A. H., Allen, E. R., and Norrell, M. A. 2012. Osteology of a North American goniopholidid (*Eutretauranosuchus delfsi*) and palate evolution in Neosuchia. *American Museum Novitates* 3783: 1-56.

Riff, D., and Kellner, A. W. A. 2011. Baurusuchid crocodyliforms as theropod mimics: clues from the skull and appendicular morphology of *Stratiotosuchus maxhechti* (Upper Cretaceous of Brazil). *Zoological Journal of the Linnean Society* 163:S37–S56.

Sereno, P. C., Larsson, H. C. E., Sidor, C. A., and Gado, B. 2001. The giant crocodyliform *Sarcosuchus* from the Cretaceous of Africa. *Science* 294:1516–1519.

Sereno, P.C., and Larsson, H. C. E. 2009. Cretaceous Crocodyliforms from the Sahara. *ZooKeys* 28:1-143.

Turner, A. H. 2006.Osteology and phylogeny of a new species of *Araripesuchus* (Crocodyliformes: Mesoeucrocodylia) from the Late Cretaceous of Madagascar. *Historical Biology* 18:255–369.

Turner, A. H., and Buckley, G. A. 2008. *Mahajangasuchus insignis* (Crocodyliformes: Mesoeucrocodylia) cranial anatomy and new data on the eusuchian–style palate. *Journal of Vertebrate Paleontology* 28:382–408.

Turner, A., and Sertich, J. J. W. 2010. Phylogenetic history of *Simosuchus clarki* (Crocodyliformes: Notosuchia) from the Late Cretaceous of Madagascar. *Journal of Vertebrate Paleontology* *Memoir* 10:177-236.

Vasconcellos F. M, and Carvalho I. S. 2010. Paleoichnological assemblage associated with Baurusuchus salgadoensis remains, a Baurusuchidae Mesoeucrocodylia from the Bauru Basin, Brazil (Late Cretaceous). *Bulletin of the New Mexico Museum of Natural History and Science* 51:227–237.

Wu, X.-C., and Sues, H.-D. 1996. Anatomy and phylogenetic relationships of *Chimaeresuchus paradoxus*, an unusual crocodyliform reptile from the Lower Cretaceous of Hubei, China. *Journal of Vertebrate Paleontology* 16:688–702.

Wu, X.-C., Sues, H.-D., and Dong, Z.-M. 1997. *Sichuanosuchus shuhanensis*: a new? Early Cretaceous protosuchian (Archosauria: Crocodyliformes) from Sichuan (China), and the monophyly of Protosuchia. *Journal of Vertebrate Paleontology* 17:89–103.

Zaher, H., Pol, D., Carvalho, A. B., Riccomini, C., Campos, D., and Nava, W. 2006. Redescription of the cranial morphology of *Mariliasuchus amarali*, and its phylogenetic affinities (Crocodyliformes, Notosuchia). *American Museum Novitates* 3512:1–40.
